# GLRX3 Acts as a [2Fe–2S] Cluster Chaperone in the Cytosolic Iron–Sulfur Assembly Machinery Transferring [2Fe–2S] Clusters to NUBP1

**DOI:** 10.1021/jacs.0c02266

**Published:** 2020-05-20

**Authors:** Francesca Camponeschi, Nihar Ranjan Prusty, Sabine Annemarie Elisabeth Heider, Simone Ciofi-Baffoni, Lucia Banci

**Affiliations:** † Magnetic Resonance Center CERM, University of Florence, Via Luigi Sacconi 6, Sesto Fiorentino, Florence 50019, Italy; ‡ Department of Chemistry, University of Florence, Via della Lastruccia 3, Sesto Fiorentino, Florence 50019, Italy

## Abstract

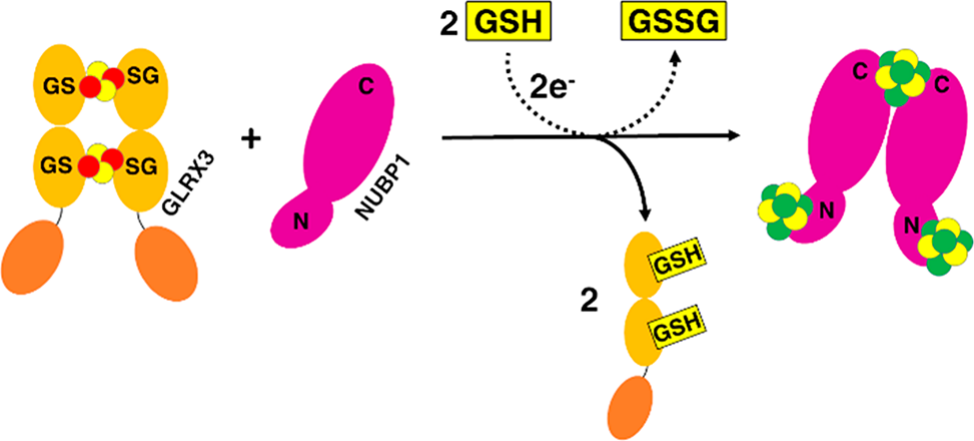

Human cytosolic monothiol glutaredoxin-3 (GLRX3) is a protein essential for the maturation of cytosolic [4Fe–4S] proteins. We show here that dimeric cluster-bridged GLRX3 transfers its [2Fe–2S]^2+^ clusters to the human P-loop NTPase NUBP1, an essential early component of the cytosolic iron–sulfur assembly (CIA) machinery. Specifically, we observed that [2Fe–2S]^2+^ clusters are transferred from GLRX3 to monomeric apo NUBP1 and reductively coupled to form [4Fe–4S]^2+^ clusters on both N-terminal CX_13_CX_2_CX_5_C and C-terminal CPXC motifs of NUBP1 in the presence of glutathione that acts as a reductant. In this process, cluster binding to the C-terminal motif of NUBP1 promotes protein dimerization, while cluster binding to the N-terminal motif does not affect the quaternary structure of NUBP1. The cluster transfer/assembly process is not complete on both N- and C-terminal motifs and indeed requires a reductant stronger than GSH to increase its efficiency. We also showed that the [4Fe–4S]^2+^ cluster formed at the N-terminal motif of NUBP1 is tightly bound, while the [4Fe–4S]^2+^ cluster bound at the C-terminal motif is labile. Our findings provide the first evidence for GLRX3 acting as a [2Fe–2S] cluster chaperone in the early stage of the CIA machinery.

## Introduction

The biogenesis of iron–sulfur (Fe–S) proteins is a highly conserved, multistep process, which involves dedicated machineries.^1^ In eukaryotes, two distinct machineries are required for the maturation of mitochondrial, cytosolic, and nuclear Fe–S proteins.^2^ In the current working model, a mitochondrial iron–sulfur cluster (ISC) assembly machinery de novo synthesizes a [2Fe–2S] cluster and then incorporates it into mitochondrial [2Fe–2S] and [4Fe–4S] target proteins.^3,4^ Another machinery in the cytosol, named cytosolic iron–sulfur assembly (CIA) machinery, is in charge of the maturation of cytosolic and nuclear [4Fe–4S] proteins.^5^ The CIA machinery has been mostly characterized in yeast, where it was proposed to begin with the assembly of a [4Fe–4S] cluster on a scaffold complex formed by two homologous P-loop nucleoside triphosphatases (NTPases) named Nbp35 and Cfd1.^6−8^ It was also proposed that the latter process is assisted by a cytosolic electron transfer chain comprising the CIA components diflavin oxidoreductase Tah18 and the Fe–S binding protein Dre2.^9^ In humans, this first phase of the CIA machinery involves the same set of proteins, which were proposed to perform the same function as their yeast homologues. Both in vivo and in vitro data indicated indeed that a [4Fe–4S] cluster assembly occurs on a scaffold complex formed by the human homologues of yeast Nbp35 and Cfd1, that is, NUBP1 and NUBP2,^10,11^ and that this process is again assisted by an electron transfer chain composed by the human homologues of yeast Tah18 and Dre2, that is, NDOR1 and anamorsin.^9^

NUBP1 and NUBP2 share a high degree of sequence identity with Nbp35 and Cfd1, respectively. While the two yeast proteins are known to form homodimeric and heterodimeric/-tetrameric complexes able to bind up to a maximum of three [4Fe–4S] clusters per dimer,^6,8,12,13^ the cluster binding properties and the quaternary structure of NUBP1 and NUBP2 proteins are not so well characterized. We only know that they form a heterocomplex in vivo and that NUBP1 binds the [4Fe–4S] cluster(s),^10^ but the biophysical data so far available do not permit one to decide how the cluster(s) are associated with NUBP1 and NUBP2. Both NUBP1 and NUBP2 have a CPXC motif in their C-terminal region as present in Nbp35 and Cfd1. This motif was found to be essential for the function of Cfd1 and of Nbp35 in the assembly of cytosolic [4Fe–4S] proteins.^12^ It coordinates a labile [4Fe–4S] cluster bridging two protein molecules in homodimeric Nbp35, in homodimeric Cfd1, and in the Cfd1–Nbp35 heterocomplex.^8,12^ Furthermore, NUBP1 and Nbp35 share a conserved N-terminal CX_13_CX_2_CX_5_C motif.^6,10,14^ In Nbp35, this motif was found to be essential for the protein function and to tightly bind a [4Fe–4S] cluster.^12^

How the assembly of the [4Fe–4S] clusters at both N- and C-terminal motifs of the two human NTPases occurs in the cytoplasm is still elusive. In particular, the source of iron and of the sulfide provided to the NUBP1–NUBP2 heterocomplex to assemble a [4Fe–4S] cluster is still a matter of debate.^2,15^ Functional data clearly showed that, in yeast, Fe–S cluster assembly on Cfd1 and Nbp35 is independent of the proteins acting later in the CIA machinery.^8^ A possible player of the CIA machinery responsible for the maturation of the [4Fe–4S] clusters on Cfd1 and Nbp35 is the yeast cytosolic monothiol glutaredoxin Grx3, which is, indeed, a crucial component for the assembly of cytosolic Fe–S proteins.^16^ In support of this model, in vivo data showed that Grx3 and its yeast paralog Grx4 bind a Fe–S cluster independent of the CIA machinery.^16^ The human proteome contains only one monothiol glutaredoxin in the cytosol, GLRX3 (also commonly named PICOT), which is essential for the maturation of cytosolic [4Fe–4S] proteins.^17^ GLRX3 has been shown to be involved in the CIA machinery by transferring its [2Fe–2S] clusters to the CIA component anamorsin, de facto acting as a [2Fe–2S] cluster chaperone in the cytosol.^18−20^ Possibly, GLRX3 could transfer its [2Fe–2S] cargo to other targets, and not only to anamorsin. However, up to now, whether GLRX3 provides [2Fe–2S] clusters to NUBP1 and NUBP2 for assembling [4Fe–4S] clusters is still unknown.

In this work, we investigated, through various spectroscopic techniques, the ability of human GLRX3 to act as a [2Fe–2S] cluster chaperone for NUBP1. We found that GLRX3 transfers its [2Fe–2S]^2+^ clusters to NUBP1. The [2Fe–2S]^2+^ clusters are received by both N-terminal and C-terminal motifs and then converted into [4Fe–4S]^2+^ clusters in the presence of glutathione (GSH), which acts as the reductant. These findings provide the first evidence for GLRX3 acting as [2Fe–2S] cluster chaperone and assembling [4Fe–4S] clusters on NUBP1.

## Experimental Section

### Cloning, Overexpression, and Purification of wtNUBP1, NUBP1_38–320_, and NUBP1-C235A/C238A Mutant in Their Apo and Holo Forms

The gene coding for human NUBP1 (UniProtKB/Swiss-Prot: P53384), inserted into the pUC-57 plasmid, was acquired from Sigma-Aldrich. The genes of wild-type NUBP1 (wtNUBP1, hereafter) and of a NUBP1 construct restricted to residues 38–320, containing only the C-terminal motif (NUBP1_38–320_, hereafter), were amplified by PCR and inserted into the pETDuet-1 expression vector using *Eco*RI and *Hin*dIII Fastdigest restriction enzymes (ThermoFisher Scientific). The NUBP1-C235A/C238A mutant was obtained through site-directed mutagenesis (Agilent QuikChange II site-directed mutagenesis kit) performed on pETDuet-wtNUBP1 according to the producer’s manual. His_6_-tagged wtNUBP1 and His_6_-tagged NUBP1-C235A/C238A were overexpressed in BL21(DE3) and His_6_-tagged NUBP1_38–320_ in BL21(DE3) Codon Plus RIPL competent *Escherichia coli* cells (Novagen). Cells were grown in Luria–Bertani containing 1 mM ampicillin at 37 °C under vigorous shaking up to a cell OD_600_ of 0.6. Protein expression was induced by adding 0.5 mM IPTG and 0.25 mM FeCl_3_. Cells were grown overnight at 21 °C (wtNUBP1 and NUBP1-C235A/C238A) and 25 °C (NUBP1_38–320_). Cells were harvested by centrifugation at 7500*g* and resuspended in lysis buffer (40 mM sodium phosphate buffer pH 8.0, 400 mM NaCl, 5 mM imidazole, containing 0.01 mg/mL DNAase, 0.01 mg/mL lysozyme, 1 mM MgSO_4_, and 5 mM dithiothreitol (DTT)). Cell disruption was performed on ice by sonication, and the soluble extract was obtained by ultracentrifugation at 40 000*g*. The following purification steps were performed aerobically to obtain the apo protein, while in an anaerobic chamber (O_2_ < 1 ppm) to isolate the protein in its holo form. The soluble fraction was loaded on a HisTrap FF column (GE Healthcare). The protein was eluted with 40 mM sodium phosphate buffer pH 8.0, 400 mM NaCl, and 400 mM imidazole, concentrated with Amicon Ultra-15 Centrifugal Filter Units with a MWCO of 10 kDa (Millipore), and the buffer was exchanged by a PD-10 desalting column in 40 mM sodium phosphate buffer pH 8.0, 400 mM NaCl, 5 mM imidazole. When required, cleavage of the His_6_ tag was performed by TEV protease overnight at room temperature. The protein mixture was then loaded on a HisTrap FF column (GE Healthcare) to separate the digested protein from the His_6_ tag.

The apo protein in the monomeric state was obtained by incubating overnight the aerobically purified protein at room temperature in 25 mM MOPS, 100 mM pyridine-2,6-dicarboxylic acid, at pH 7.0.

Chemical reconstitution was performed inside an anaerobic chamber (O_2_ < 1 ppm), by incubating overnight at room temperature the monomeric apo protein in degassed 50 mM Tris, 100 mM NaCl, 5 mM DTT, at pH 8.0 with up to a 12-fold excess of FeCl_3_ and Na_2_S. Excess of FeCl_3_ and Na_2_S was anaerobically removed by passing the mixture on a PD-10 desalting column, and the holo protein was recovered.

### Production of [2Fe–2S]_2_-GLRX3_2_-GS_4_

GLRX3 was expressed, purified, and chemically reconstituted following previously reported procedures.^18^

### Protein, Iron, and Acid-Labile Sulfide Quantification

Protein quantification was carried out with the Bradford protein assay, using BSA as a standard. Nonheme iron and acid-labile sulfide content was determined as described previously.^21^

### Biochemical and Spectroscopic UV–Vis, CD, EPR, and NMR Methods

The quaternary structure of the proteins was analyzed through analytical gel filtration on a Superdex 200 10/300 Increase column (GE Healthcare). Column was calibrated with a gel filtration marker calibration kit, 6500–66 000 Da (Sigma-Aldrich), to obtain the apparent molecular masses of the detected species. Samples in degassed 50 mM phosphate buffer pH 7.0, 200 mM NaCl, 5 mM DTT (plus 5 mM GSH for [2Fe–2S]_2_-GLRX3_2_-GS_4_), were loaded on the pre-equilibrated column. Elution profiles were recorded at 280 nm with a flow rate of 0.65 mL/min.

UV–visible (UV–vis) spectra were anaerobically acquired on a Cary 50 Eclipse spectrophotometer in degassed 50 mM phosphate buffer pH 7.0 (plus 5 mM GSH for [2Fe–2S]_2_-GLRX3_2_-GS_4_).

Circular dichroism (CD) spectra were acquired on a JASCO J-810 spectropolarimeter in 20 mM phosphate buffer pH 7.0.

CW EPR spectra were recorded before and after the anaerobic reduction of the cluster(s) by addition of up to 5 mM sodium dithionite. Protein concentration was in the range 0.5–0.7 mM, in degassed 50 mM Tris buffer pH 8.0, 100 mM NaCl, 5 mM DTT, and 10% glycerol. EPR spectra were acquired at 10 and 45 K, using a Bruker Elexsys 580 spectrometer working at a microwave frequency of ca. 9.36 GHz, equipped with a SHQ cavity and a continuous flow He cryostat (ESR900, Oxford instruments) for temperature control. Acquisition parameters were as follows: microwave frequency, 9.36 GHz; microwave power, 1 mW at 10 K and 0.12 mW at 45 K; modulation frequency, 100 kHz; modulation amplitude, 10 G; acquisition time constant, 163.84 ms; number of points 1024; number of scans 4; field range 2000–4000 G.

Paramagnetic 1D ^1^H NMR experiments were performed on a Bruker Avance spectrometer operating at 400 MHz ^1^H Larmor frequency and equipped with a ^1^H dedicated 5 mm probe. Water signal was suppressed via fast repetition experiments and water selective irradiation.^22^ Experiments were typically performed using an acquisition time of 50 ms and an overall recycle delay of 80 ms. Sample concentration was in the range 0.5–0.7 mM, in degassed 50 mM phosphate buffer pH 7.0. Squared cosine and exponential multiplications were applied prior to Fourier transformation. Manual baseline correction was performed using polynomial functions.

### Cluster Transfer from [2Fe–2S]_2_-GLRX3_2_-GS_4_ to His_6_-Tagged Apo wtNUBP1, His_6_-Tagged Apo NUBP1_38–320_, and His_6_-Tagged Apo NUBP1-C235A/C238A Mutant

Each His_6_-tagged monomeric apo NUBP1 species was incubated under anaerobic conditions with different amounts of [2Fe–2S]_2_-GLRX3_2_-GS_4_, depending on the number of cluster binding motifs contained in each NUBP1 species. Specifically, His_6_-tagged monomeric apo wtNUBP1 was incubated with 0.50, 1.0, and 1.5 equiv of [2Fe–2S]_2_-GLRX3_2_-GS_4_, His_6_-tagged monomeric apo NUBP1-C235A/C238A was incubated with 0.3, 0.6, and 1.0 equiv of [2Fe–2S]_2_-GLRX3_2_-GS_4_, and His_6_-tagged monomeric apo NUBP1_38–320_ was incubated with 0.15, 0.3, and 0.50 equiv of [2Fe–2S]_2_-GLRX3_2_-GS_4_, all in the presence of 5 mM GSH, for 1 h at room temperature in 40 mM sodium phosphate buffer pH 8.0, 400 mM NaCl, and 5 mM imidazole. The final ratios of 1.5:1.0, 1.0:1.0, and 0.5:1.0 correspond to the stoichiometric amounts of [2Fe–2S]_2_-GLRX3_2_-GS_4_ required to fully saturate the cluster binding motifs present in the three proteins with a [4Fe–4S] cluster, that is, three per dimeric wtNUBP1, one per monomeric NUBP1-C235A/C238A, and one per dimeric NUBP1_38–320_. Separation of the His_6_-tagged NUBP1 species from untagged GLRX3 after reaction was performed in anaerobic conditions by loading the reaction mixtures on a His GraviTrap column pre-equilibrated with 40 mM sodium phosphate buffer pH 8.0, 400 mM NaCl, and 5 mM imidazole. The His_6_-tagged NUBP1 species was eluted with 40 mM sodium phosphate buffer pH 8.0, 400 mM NaCl, and 400 mM imidazole. After concentration, the buffer was exchanged by a PD-10 desalting column in the appropriate degassed buffer required to perform analytical gel filtration, iron and acid-labile sulfide quantification, and to acquire UV–vis and paramagnetic 1D ^1^H NMR spectra.

### Cluster Transfer from [2Fe–2S]_2_-GLRX3_2_-GS_4_ to His_6_-Tagged Apo wtNUBP1 in the Presence of Different GSH Concentrations

His_6_-tagged monomeric apo wtNUBP1 was incubated under anaerobic conditions with 1.5 equiv of [2Fe–2S]_2_-GLRX3_2_-GS_4_, in the presence of increasing amounts of GSH (0, 1, 5, and 10 mM), for 1 h at room temperature in 40 mM sodium phosphate buffer pH 8.0, 400 mM NaCl, and 5 mM imidazole. Separation of His_6_-tagged wtNUBP1 from untagged GLRX3 after reaction was performed in anaerobic conditions as described above. After concentration, the buffer was exchanged by a PD-10 desalting column in degassed 50 mM phosphate buffer pH 7.0. and UV–vis spectra were then acquired.

## Results

### Fe–S Cluster Binding Properties and Quaternary Structure of Human NUBP1

**Figure 1:**
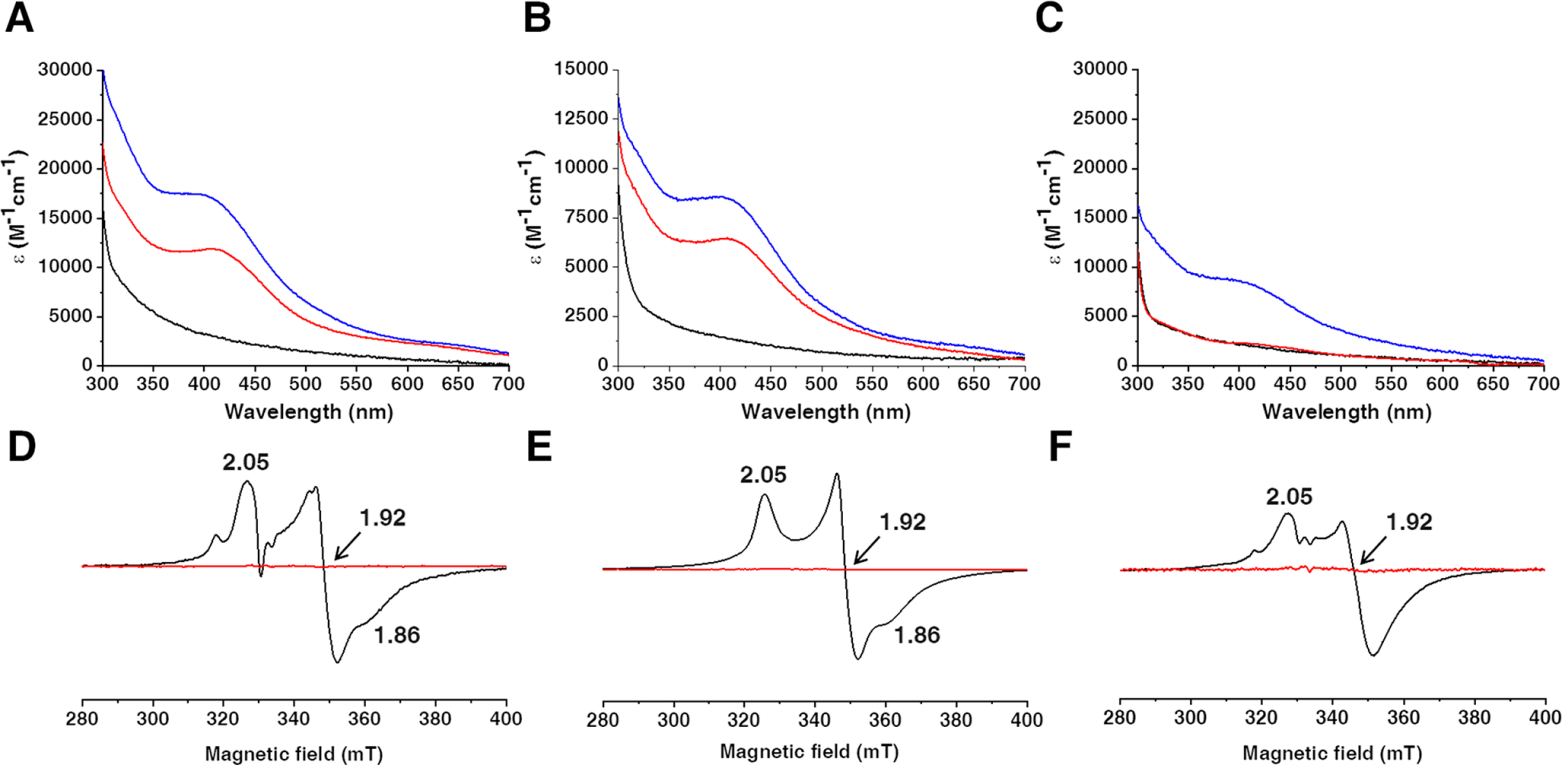
NUBP1 binds [4Fe–4S]^2+^ clusters at both N-terminal and C-terminal motifs. UV–vis spectra of (A) wtNUBP1, (B) NUBP1-C235A/C238A, and (C) NUBP1_38–320_, aerobically purified (black line), anaerobically purified (red line), and chemically reconstituted (blue line). ε values are based on monomeric (NUBP1-C235A/C238A) or dimeric protein (wtNUBP1 and NUBP1_38–320_) concentration. CW X-band EPR spectra of anaerobically purified wtNUBP1 (D), anaerobically purified NUBP1-C235A/C238A (E), and chemically reconstituted NUBP1_38–320_ (F), after reduction with sodium dithionite, at 10 K (black line) and at 45 K (red line).

The characterization of the Fe–S cluster binding properties and quaternary structure of NUBP1 is an essential prerequisite to investigate in detail Fe–S cluster transfer from GLRX3 to NUBP1. For this purpose, in addition to wild-type NUBP1 (wtNUBP1, hereafter), we produced a construct where the first 37 N-terminal residues were deleted (NUBP1_38–320_, hereafter), which thus contains only the C-terminal CPXC motif, and a mutant containing only the N-terminal CX_13_CX_2_CX_5_C motif, by mutating the two cysteines of the CPXC motif, that is, C235 and C238, into alanines (NUBP1-C235A/C238A, hereafter). The N-terminal deletion of NUBP1 as well as C235A/C238A mutations did not affect protein folding, as shown by circular dichroism and 1D ^1^H NMR spectroscopy (Figure S1). UV–vis spectra of anaerobically purified wtNUBP1 and NUBP1-C235A/C238A exhibit a broad absorption band at ∼410 nm, which is characteristic of a [4Fe–4S]^2+^ cluster^23^ (Figure 1A and B). The samples are EPR silent, as expected for the *S* = 0 ground state of an oxidized [4Fe–4S]^2+^ cluster.^24^ Chemical reduction with sodium dithionite of both samples gave rise to similar intense rhombic EPR signals at 10 K, with *g* values of 2.05, 1.92, and 1.86, which broadened beyond detection at 45 K (Figure 1D and E), in agreement with the presence of [4Fe–4S]^+^ clusters. These results compare quite well with what was previously observed for the yeast NUBP1 homologue Nbp35.^8^

**Figure 2:**
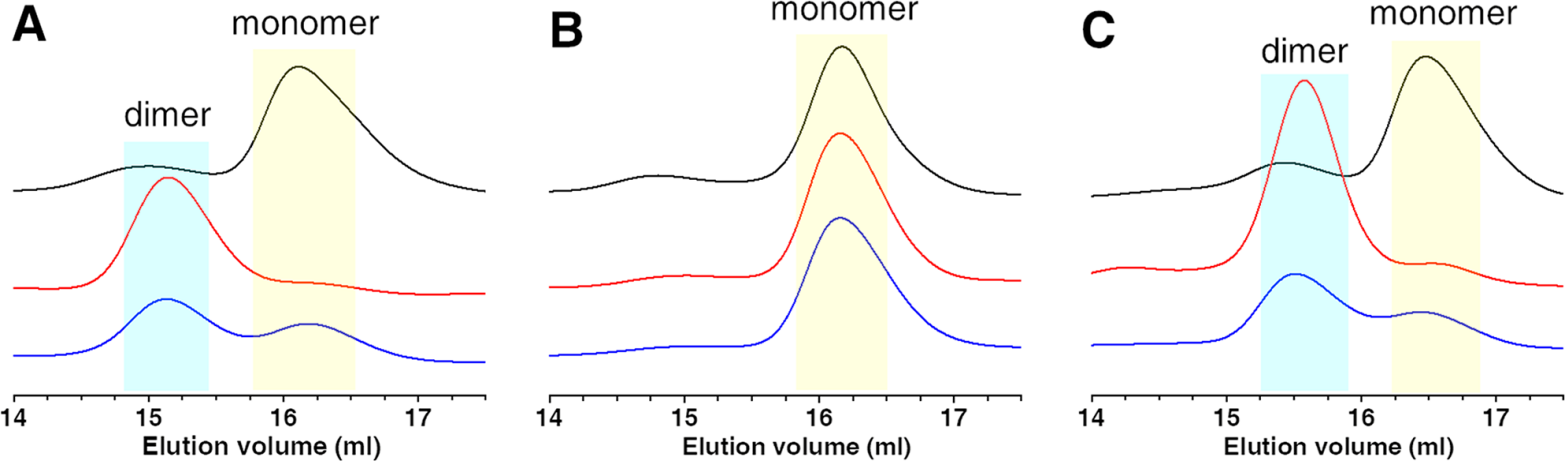
The C-terminal motif of NUBP1 promotes protein dimerization. Analytical gel filtration profiles of (A) wtNUBP1, (B) NUBP1-C235A/C238A, and (C) NUBP1_38–320_, anaerobically purified (red line), chemically reconstituted from the monomeric apo form (blue line), and after treatment of the anaerobically/aerobically purified form with pyridine-2,6-dicarboxylic acid (PDC) to remove metals (black line).

Anaerobically purified wtNUBP1 and NUBP1-C235A/C238A showed differences in their quaternary structure, as determined by analytical gel filtration: wtNUBP1 is dimeric, whereas NUBP1-C235A/C238A is monomeric (Figure 2A and B). Anaerobically purified wtNUBP1 and NUBP1-C235A/C238A contained 0.9 and 0.3 [4Fe–4S] clusters per dimer and monomer, respectively, according to protein, acid-labile sulfide, and iron analysis (Table 1). These data indicate the presence of substoichiometric amounts of cluster(s) in anaerobically purified wtNUBP1 and NUBP1-C235A/C238A (0.9 vs 3 clusters and 0.3 vs 1 cluster, respectively).

**Table 1:** Iron and Acid-Labile Sulfide Quantification of Anaerobically Purified and Chemically Reconstituted Proteins

sample	Fe^a^	S^a^	[4Fe–4S]
wtNUBP1* anaerobically purified	3.6 ± 0.1	3.6 ± 0.1	0.90
wtNUBP1* chemically reconstituted	5.3 ± 0.1	5.1 ± 0.1	1.30
NUBP1-C235A/C238A** anaerobically purified	1.2 ± 0.1	1.2 ± 0.1	0.30
NUBP1-C235A/C238A** chemically reconstituted	3.1 ± 0.1	3.0 ± 0.1	0.80
NUBP1_38–320_* anaerobically purified	0.2 ± 0.1	0.1 ± 0.1	0.04
NUBP1_38–320_* chemically reconstituted	2.2 ± 0.1	2.0 ± 0.1	0.50

a Fe and acid-labile S measurements are reported as mol Fe or S per mol of dimeric* or monomeric** protein. Data are the average of three independent samples.

**Figure 3:**
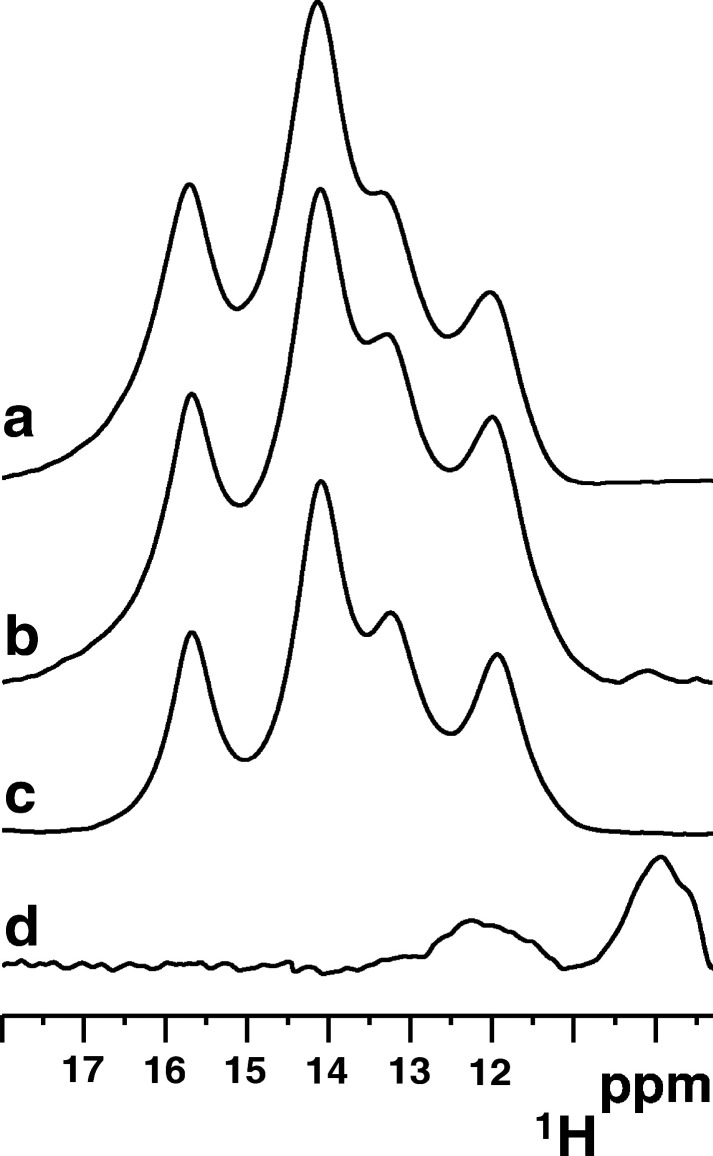
Paramagnetic NMR spectroscopy showed that NUBP1 binds [4Fe–4S]^2+^ clusters. Paramagnetic 1D ^1^H NMR spectra of (a) anaerobically purified and (b) chemically reconstituted wtNUBP1, (c) anaerobically purified NUBP1-C235A/C238A, and (d) chemically reconstituted NUBP1_38–320_.

Paramagnetic 1D ^1^H NMR spectra of these samples showed four intense hyperfine shifted signals in the 18–11 ppm spectral region (Figure 3, traces a and c), whose chemical shift values and line widths are typical of βCH_2_ of cysteines bound to a [4Fe–4S]^2+^ cluster.^25,26^ The anti-Curie temperature dependence of these signals further confirms the presence of an oxidized [4Fe–4S]^2+^ cluster (Figure S2). Moreover, the observation that the paramagnetic 1D ^1^H NMR spectrum of anaerobically purified wtNUBP1 is well superimposable to that of anaerobically purified NUBP1-C235A/C238A (Figure 3), which can only bind a [4Fe–4S]^2+^ cluster at the N-terminal motif, indicates that the [4Fe–4S]^2+^ clusters in the anaerobically purified wtNUBP1 are bound to the N-terminal motif, and thus the occupancy of the C-terminal site by [4Fe–4S]^2+^ clusters is very low.

Contrary to wtNUBP1 and NUBP1-C235A/C238A, anaerobically purified NUBP1_38–320_ was essentially colorless, contained less than 5% of [4Fe–4S] cluster (Table 1), and showed no signals in the UV–vis spectrum (Figure 1C) as well as in the paramagnetic 1D ^1^H NMR and EPR spectra, the latter even after dithionite reduction (data not shown), indicating that it does not bind Fe/S clusters, as was previously observed in a truncated form of Nbp35 lacking the N-terminal 52 amino acids.^8^ Anaerobically purified NUBP1_38–320_ is homodimeric, as determined by analytical gel filtration (Figure 2C). Incubation of anaerobically purified NUBP1_38–320_ as well as of anaerobically and aerobically purified wtNUBP1, which are both totally in a dimeric state, although they contain from zero to substoichiometric percentages of bound Fe/S clusters, with pyridine-2,6-dicarboxylic acid (PDC), a strong divalent transition metal chelator, led to their monomerization (Figure 2A and C). This indicates that their dimerization is promoted, not only by the binding of a bridged Fe/S cluster, but also by the binding of an adventitious metal ion(s), shared by C235 and/or C238 from each subunit of the dimer. Consistent with this behavior, the treatment of both anaerobically and aerobically purified NUBP1-C235A/C238A with PDC does not affect the quaternary structure that is always monomeric (Figure 2B). Upon chemical reconstitution of monomerized apo wtNUBP1, monomeric apo NUBP1-C235A/C238A, and monomerized apo NUBP1_38–320_ species in the presence of a 12-fold excess of FeCl_3_ and Na_2_S, under reductive, anaerobic conditions, the broad absorption band at ∼410 nm, typical of oxidized [4Fe–4S]^2+^ protein-bound clusters, appeared in the UV–vis spectra of NUBP1_38–320_, wtNUBP1, and NUBP1-C235A/C238A, having, in the latter two, higher intensity with respect to that in the UV–vis spectra of the anaerobically purified proteins (Figure 1). After chemical reconstitution, the quantification of iron and acid-labile sulfide indicated that ∼1.3, ∼0.8, and ∼0.5 of [4Fe–4S] clusters are bound per dimeric wtNUBP1, per monomeric NUBP1-C235A/C238A, and per dimeric NUBP1_38–320_, respectively (Table 1). These data indicate that chemical reconstitution of the monomeric/monomerized apo proteins is able to load, with [4Fe–4S]^2+^ clusters, both C- and N-terminal motifs, even though the chemical reconstitution efficiency was partial. Specifically, the iron and acid-labile sulfide quantification data on the two mutants showed that the [4Fe–4S]^2+^ cluster occupancy at the N-terminal site is higher than that of the C-terminal site, suggesting an intrinsic lability of the [4Fe–4S]^2+^ cluster binding at the C-terminal site.

The lability of the [4Fe–4S] cluster binding at the C-terminal site was further suggested by the different behaviors of [4Fe–4S] NUBP1_38–320_ and [4Fe–4S] NUBP1 C235A/C238A during the acquisition of 1D ^1^H paramagnetic NMR experiments. The latter were acquired, in the presence of 5 mM DTT and in anaerobic conditions, as a series of 1 h spectra, over a period of roughly 12 h. Over this period of time, the intensity of the paramagnetic signals of [4Fe–4S] NUBP1_38–320_ decreased, while that of the signals of [4Fe–4S] NUBP1 C235A/C238A did not, clearly indicating cluster loss from the former mutant. To further corroborate the latter proposal, a freshly prepared sample of chemically reconstituted NUBP1_38–320_ was anaerobically sealed in a quartz cuvette and in the presence of 5 mM DTT, to completely avoid cluster oxidation over time, and UV–vis spectra were recorded for 24 h. It resulted that the band at ∼410 nm characteristic of a [4Fe–4S]^2+^ cluster decreases in intensity (Figure S3), indicating that the [4Fe–4S] cluster bound to the C-terminal motif is kinetically labile. The same behavior was not observed for the chemically reconstituted NUBP1-C235A/C238A, which, indeed, does not lose the cluster over time. A freshly prepared chemically reconstituted NUBP1_38–320_ was anaerobically reduced by addition of sodium dithionite and rapidly frozen. The EPR spectrum, acquired at 10 K, showed an axial signal with *g* values of 2.05 and 1.92 that broadened beyond detection at 45 K (Figure 1F), in agreement with the presence of a [4Fe–4S]^+^ cluster bound to the C-terminal motif. This spectrum is similar to that previously reported for a construct of Nbp35 lacking the N-terminal motif.^8^

Analytical gel filtration of the species obtained chemically reconstituting the monomeric proteins showed that dimerization occurred for wtNUBP1 and NUBP1_38–320_, but not for NUBP1-C235A/C238A (Figure 2). Specifically, wtNUBP1 and NUBP1_38–320_ were eluted in two fractions: the one eluting at smaller elution volume (at ∼15 mL), that is, the dimeric species, has a dark brown color and thus contains a dimeric [4Fe–4S]^2+^ NUBP1 species, while the fraction eluting at ∼16 mL, that is, the monomeric species, is colorless and thus contains the apo protein. This result indicates that cluster binding to the C-terminal motif induces protein dimerization by bridging two C-terminal motifs, as was previously observed in yeast Nbp35.^12^ Moreover, the presence of the monomeric apo species in the gel filtration profiles of the chemically reconstituted monomerized apo proteins (Figure 2) is in agreement with the partial cluster loading resulting from the iron and acid-labile sulfide quantification data (Table 1). Taking into account the molar fractions of holo wtNUBP1 versus the apo form in the chemically reconstituted sample (i.e., 0.6 and 0.4, respectively, estimated from the gel filtration profile, Figure 2A) and the cluster content (Table 1), we estimated that ∼2.2 of [4Fe–4S] clusters were bound to the fraction of dimeric wtNUBP1, indicating that the three cluster binding sites in the dimeric holo wtNUBP1 species, that is, two N-terminal and one C-terminal, are partially occupied by [4Fe–4S] clusters. Considering the observed kinetic lability of the [4Fe–4S] cluster binding to the C-terminal motif, it is reasonable to conclude that, in the dimeric chemically reconstituted holo wtNUBP1 species, the two N-terminal cluster binding sites are essentially fully occupied by [4Fe–4S] clusters, while the C-terminal cluster binding site is occupied at very low extent, that is, ∼20% occupied. This model is supported by the paramagnetic 1D ^1^H NMR spectra of freshly prepared chemically reconstituted NUBP1_38–320_ and wtNUBP1. The first showed very weak hyperfine shifted signals at 12 and 10 ppm in the spectral region typical of βCH_2_ of cysteines bound to a [4Fe–4S]^2+^ cluster (Figure 3, trace d), and their very low signal intensity agrees with a very low [4Fe–4S] cluster occupancy at the C-terminal site, as a consequence of the [4Fe–4S]^2+^ cluster binding lability at this motif. The second showed very intense hyperfine shifted signals of cysteines bound to the N-terminal cluster, and a very weak hyperfine shifted signal at 10 ppm diagnostic of a C-terminal bound cluster, which is indicative of a low [4Fe–4S] cluster occupancy at the C-terminal site (Figure 3, trace b). This signal was not observed in anaerobically purified wtNUBP1 (Figure 3, trace a), which confirms that the C-terminal site was not occupied by [4Fe–4S] clusters in the latter sample.

In conclusion, wtNUBP1 binds three [4Fe–4S]^2+^ clusters, two, as stable, at each N-terminal CX_13_CX_2_CX_5_C motif, and one at the C-terminal CPXC motif, which is labile and essential for promoting protein dimerization. This behavior is the same as that observed for the yeast homologue of NUBP1, that is, Nbp35.^8^

### [2Fe–2S]_2_-GLRX3_2_-GS_4_ Transfers Its Clusters to NUBP1

GLRX3 consists of three domains: one N-terminal thioredoxin domain and two monothiol glutaredoxin domains, each able to bind a glutathione-coordinated [2Fe–2S]^2+^ cluster via protein dimerization ([2Fe–2S]_2_-GLRX3_2_-GS_4_, hereafter).^27−29^ Various amounts of [2Fe–2S]_2_-GLRX3_2_-GS_4_ were incubated with monomerized apo wtNUBP1, or with monomeric apo NUBP1-C235A/C238A, or with monomerized apo NUBP1_38–320_ under anaerobic conditions and in the presence of 5 mM GSH, and, for each experiment, the two proteins in the mixture were separated through nickel-affinity chromatography thanks to the presence of a His_6_-tag on NUBP1 (see the Experimental Section for details). The cluster transfer/assembly events were then monitored by UV–vis and paramagnetic 1D ^1^H NMR spectra, by performing acid-labile sulfide and iron quantification and analytical gel filtration on the isolated proteins before and after their mixing. Considering the high complexity of a mechanism possibly involving cluster transfer and/or assembly on the N-terminal and C-terminal motifs of wtNUBP1, we have first investigated the transfer to NUBP1-C235A/C238A and to NUBP1_38–320_ mutants that hold just one cluster binding site each.

**Figure 4:**
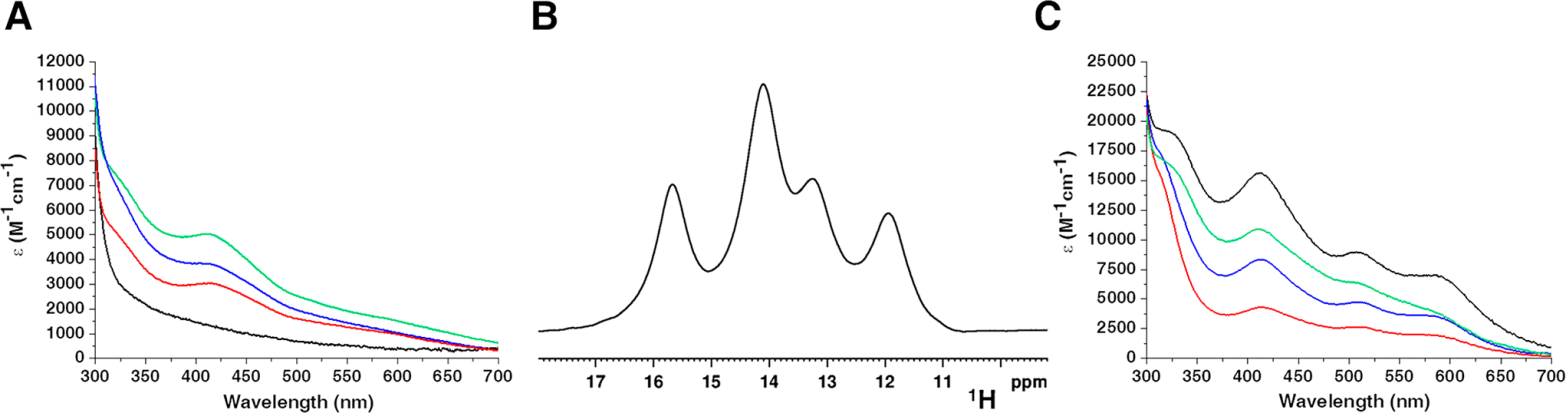
[2Fe–2S]^2+^ clusters are transferred from [2Fe–2S]_2_-GLRX3_2_-GS_4_ to NUBP1-C235A/C238A and reductively coupled to form a [4Fe–4S]^2+^ cluster. (A) UV–vis spectra of NUBP1-C235A/C238A before (black line) and after incubation with 0.3 equiv (red line), 0.6 equiv (blue line), and 1.0 equiv (green line) of [2Fe–2S]_2_-GLRX3_2_-GS_4_. (B) Paramagnetic 1D ^1^H NMR spectrum of NUBP1-C235A/C238A obtained after reaction with 1 equiv of [2Fe–2S]_2_-GLRX3_2_-GS_4_ in the presence of 5 mM GSH. (C) UV–vis spectra of chemically reconstituted [2Fe–2S]_2_-GLRX3_2_-GS_4_ before (black line) and after incubation with NUBP1-C235A/C238A at the 0.3:1.0 (red line), 0.6:1.0 (blue line), and 1.0:1.0 (green line) [2Fe–2S]_2_-GLRX3_2_-GS_4_:NUBP1-C235A/C238A stoichiometric ratios. ε values are based on the monomeric protein (NUBP1-C235A/C238A) or dimeric protein ([2Fe–2S]_2_-GLRX3_2_-GS_4_) concentration.

Monomeric apo NUBP1-C235A/C238A was mixed with different amounts of [2Fe–2S]_2_-GLRX3_2_-GS_4_ (0.3, 0.6, and 1.0 equiv of [2Fe–2S]_2_-GLRX3_2_-GS_4_ per monomeric apo NUBP1-C235A/C238A, with 1 equiv being the stoichiometric amount required to form one [4Fe–4S] cluster on monomeric NUBP1-C235A/C238A), and the UV–vis spectra of the isolated NUBP1-C235A/C238A protein showed that an absorbance band at ∼410 nm, characteristic of the oxidized [4Fe–4S]^2+^ cluster-bound form of NUBP1-C235A/C238A, appeared and increased in intensity upon increasing the [2Fe–2S]_2_-GLRX3_2_-GS_4_ amount (Figure 4A). Four intense hyperfine shifted signals were observed in the paramagnetic 1D ^1^H NMR spectrum of NUBP1-C235A/C238A isolated from the reaction with 1 equiv of [2Fe–2S]_2_-GLRX3_2_-GS_4_ (Figure 4B). These signals are the same as those observed in the paramagnetic 1D ^1^H NMR spectrum of [4Fe–4S]^2+^ NUBP1-C235A/C238A (compare Figure 4B with Figure 3, trace c).

The cluster transfer/assembly reaction is relatively fast, as it occurs within 1 h after the mixing of the two proteins. Acid-labile sulfide and iron analysis on NUBP1-C235A/C238A, isolated after incubation with 1 equiv of [2Fe–2S]_2_-GLRX3_2_-GS_4_, showed that NUBP1-C235A/C238A contains 0.5 [4Fe–4S] clusters per monomer (Table 2), consistent with a [4Fe–4S] cluster assembly efficiency of ∼60% with respect to chemical reconstitution. The absorbance peaks in the UV–vis spectra of [2Fe–2S]_2_-GLRX3_2_-GS_4_, once incubated with apo NUBP1-C235A/C238A and then isolated, were significantly reduced with respect to the starting material (Figure 4C). However, their intensities were not completely bleached at any protein ratio, being higher as they went from substoichiometric ratios to the 1:1 ratio (Figure 4C). This result indicates no complete cluster transfer/assembly reaction. An excess of [2Fe–2S]_2_-GLRX3_2_-GS_4_ (2.5 equiv of [2Fe–2S]_2_-GLRX3_2_-GS_4_ per monomeric apo NUBP1-C235A/C238A) was not able to increase the [4Fe–4S] incorporation into NUBP1-C235A/C238A; that is, the absorbance band at ∼410 nm did not increase in intensity in the isolated NUBP1-C235A/C238A protein. Acid-labile sulfide and iron analysis on the residual [2Fe–2S]_2_-GLRX3_2_-GS_4_, isolated after incubation with 1 equiv of NUBP1-C235A/C238A, showed that [2Fe–2S]_2_-GLRX3_2_-GS_4_ contains ∼0.9 [2Fe–2S] clusters per dimer (Table 2). These data indicate that ∼50% of [2Fe–2S] clusters were released from [2Fe–2S]_2_-GLRX3_2_-GS_4_ at the 1:1 ratio, corresponding to the formation of ∼0.5 [4Fe–4S] clusters. NUBP1-C235A/C238A isolated from the same mixture contains, indeed, 0.5 [4Fe–4S] clusters, indicating that all of the [2Fe–2S] clusters released from [2Fe–2S]_2_-GLRX3_2_-GS_4_ are coordinated to NUBP1 with the formation of [4Fe–4S] clusters, and thus no cluster is lost in solution in the cluster transfer/assembly process. These observations suggest that, upon addition of [2Fe–2S]_2_-GLRX3_2_-GS_4_, there is a transfer of [2Fe–2S] clusters to NUBP1 with the concomitant assembly of [4Fe–4S] clusters, although this process is not complete.

**Table 2:** Iron and Acid-Labile Sulfide Quantification of [2Fe–2S]_2_-GLRX3_2_-GS_4_, wtNUBP1, NUBP1-C235A/C238A, and NUBP1_38–320_ before and after Cluster Transfer/Assembly Reaction

sample	Fe^a^	S^a^	[2Fe–2S]	[4Fe–4S]
[2Fe–2S]_2_-GLRX3_2_-GS_4_* chemically reconstituted	3.5 ± 0.1	3.3 ± 0.1	1.7 ± 0.1	
[2Fe–2S]_2_-GLRX3_2_-GS_4_* after mixing with NUBP1-C235A/C238A (1.0:1.0)	1.8 ± 0.1	1.9 ± 0.1	0.9 ± 0.1	
[2Fe–2S]_2_-GLRX3_2_-GS_4_* after mixing with NUBP1_38–320_ (0.5:1.0)	2.3 ± 0.1	2.4 ± 0.1	1.2 ± 0.1	
[2Fe–2S]_2_-GLRX3_2_-GS_4_* after mixing with wtNUBP1 (1.5:1.0)	1.7 ± 0.1	1.8 ± 0.1	0.9 ± 0.1	
NUBP1-C235A/C238A** after mixing with [2Fe–2S]_2_-GLRX3_2_-GS_4_ (1.0:1.0)	1.9 ± 0.1	1.8 ± 0.1		0.5 ± 0.1
NUBP1_38–320_* after mixing with [2Fe–2S]_2_-GLRX3_2_-GS_4_ (1.0:0.5)	1.1 ± 0.1	1.0 ± 0.1	0.5 ± 0.1	
wtNUBP1* after mixing with [2Fe–2S]_2_-GLRX3_2_-GS_4_ (1.0:1.5)	2.5 ± 0.1	2.6 ± 0.1		0.6 ± 0.1

a Fe and acid-labile S measurements are reported as mol Fe or S per mol of dimeric* or monomeric** protein. Data are the average of three independent samples.

Addition of 5 mM of DTT, that is, a reductant stronger than GSH although not physiologically relevant,^30−32^ to the 1:1 mixture promotes the formation of [4Fe–4S] clusters as indicated by a further increase in the intensities of the band at 410 nm in the UV–vis spectra (Figure S4). At the same time, the intensity of the UV–vis signals of GLRX3, isolated after incubation with NUBP1 C235A/C238A in the presence of 5 mM DTT and separation on Ni-NTA column, is lower than that observed in the absence of DTT (Figure S4). Overall, these data indicate that, upon DTT addition, more [2Fe–2S] clusters are released from GLRX3 and concomitantly more [4Fe–4S] clusters assembled on NUBP1 C235A/C238A. This effect can be rationalized considering the higher reduction potential of DTT (*E*_pH=8_ = −366 mV)^33^ than that of GSH (*E*_pH=8_ = −299 mV),^34^ which thus can drive a more efficient reductive coupling of two [2Fe–2S]^2+^ clusters to form a [4Fe–4S]^2+^ cluster on NUBP1-C235A/C238A. In conclusion, the incomplete [4Fe–4S] cluster assembly on the N-terminal motif could be due to the fact that GSH reduction potential is not sufficient to efficiently drive [4Fe–4S] cluster formation on NUBP1 C235A/C238A, regardless of any excess of [2Fe–2S]_2_-GLRX3_2_-GS_4_ added to the mixture.

**Figure 5:**
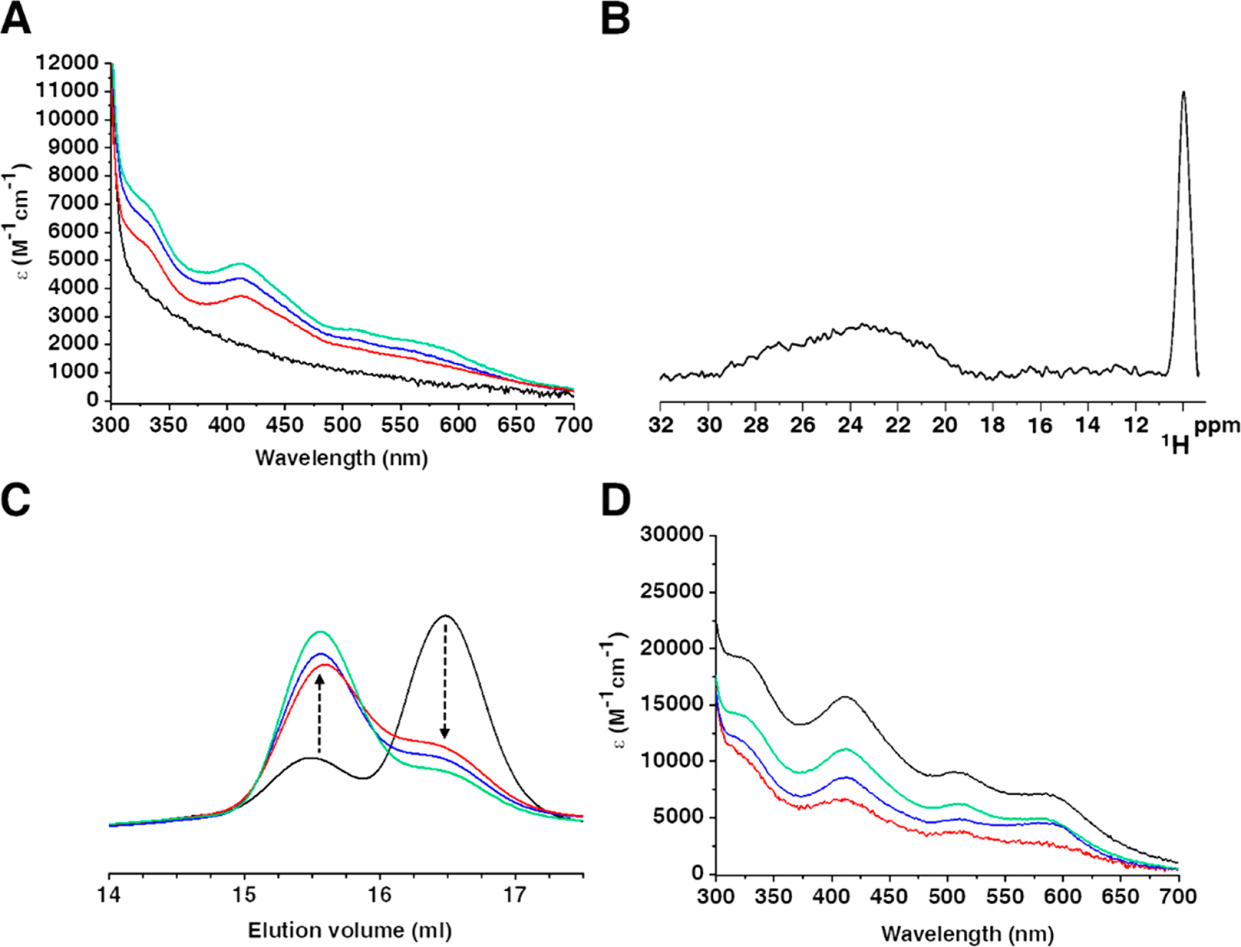
[2Fe–2S]_2_-GLRX3_2_-GS_4_ transfers a [2Fe–2S]^2+^ cluster to NUBP1_38–320_. (A) UV–vis spectra of NUBP1_38–320_ before (black line) and after incubation with 0.15 equiv (red line), 0.30 equiv (blue line), and 0.50 equiv (green line) of [2Fe–2S]_2_-GLRX3_2_-GS_4_. (B) Paramagnetic 1D ^1^H NMR spectrum of NUBP1_38–320_ obtained after reaction with 0.50 equiv of [2Fe–2S]_2_-GLRX3_2_-GS_4_ in the presence of 5 mM GSH. (C) Analytical gel filtration of NUBP1_38–320_ before (black line) and after incubation with 0.15 equiv (red line), 0.30 equiv (blue line), and 0.50 equiv (green line) of [2Fe–2S]_2_-GLRX3_2_-GS_4_. (D) UV–vis spectra of chemically reconstituted [2Fe–2S]_2_-GLRX3_2_-GS_4_ before (black line) and after incubation with NUBP1_38–320_ at the 0.15:1.0 (red line), 0.3:1.0 (blue line), and 0.5:1.0 (green line) [2Fe–2S]_2_-GLRX3_2_-GS_4_:NUBP1_38–320_ ratios. ε values are based on dimeric protein concentration.

After incubation of NUBP1_38–320_ with increasing amounts of [2Fe–2S]_2_-GLRX3_2_-GS_4_, up to the NUBP1_38–320_:[2Fe–2S]_2_-GLRX3_2_-GS_4_ 1:0.5 ratio (the latter is the stoichiometric amount required to form one [4Fe–4S] cluster on dimeric NUBP1_38–320_), the UV–vis spectrum of NUBP1_38–320_ separated from the mixture (Figure 5A) did not match that of the reconstituted [4Fe–4S]^2+^ NUBP1_38–320_ (Figure 1C), because it showed bands at 320, 510, and 580 nm, which are typical of a [2Fe–2S]^2+^ cluster.^35^ These bands increased in intensity upon increasing amounts of [2Fe–2S]_2_-GLRX3_2_-GS_4_ (Figure 5A), indicating a stepwise formation of [2Fe–2S]^2+^ NUBP1_38–320_. Even longer incubation times, up to 4 h, did not promote the formation of a [4Fe–4S] cluster on the C-terminal site. SDS-PAGE analysis allowed us to exclude the presence of traces of coeluted [2Fe–2S]_2_-GLRX3_2_-GS_4_ in the fraction containing NUBP1_38–320_ (Figure S5), thus confirming that the [2Fe–2S]^2+^ cluster observed in the UV–vis spectra of NUBP1_38–320_ separated from the mixture is exclusively bound to NUBP1_38–320_. The ^1^H NMR spectrum of NUBP1_38–320_ after incubation and separation from [2Fe–2S]_2_-GLRX3_2_-GS_4_ showed a broad unresolved signal in the 30–20 ppm spectral region and a sharper signal at 10 ppm (Figure 5B). These two signals are typical of βCH_2_ and αCH, respectively, of cysteines bound to a [2Fe–2S]^2+^ cluster.^25,36^ Analytical gel filtration data showed that cluster transfer from [2Fe–2S]_2_-GLRX3_2_-GS_4_ to monomerized apo NUBP1_38–320_ induces stepwise protein dimerization (Figure 5C). The cluster transfer/assembly reaction is relatively fast as it occurs within 1 h after the mixing of the two proteins. Acid-labile sulfide and iron analysis of NUBP1_38–320_, isolated from the 1:0.5 apo NUBP1_38–320_:[2Fe–2S]_2_-GLRX3_2_-GS_4_ mixture, showed that NUBP1_38–320_ contains ∼0.5 [2Fe–2S] clusters per dimer (Table 2), indicating an incomplete [2Fe–2S] cluster transfer reaction at the [2Fe–2S]_2_-GLRX3_2_-GS_4_ stoichiometric amount required to fully saturate NUBP1_38–320_ with a [4Fe–4S] cluster. The observed partial cluster transfer could be possibly due to similar [2Fe–2S] cluster binding affinities between the C-terminal motif of NUBP1_38–320_ and the two CGFS motifs of GLRX3. Consistent with the observed partial cluster transfer, the absorbance peaks of the [2Fe–2S]^2+^ clusters of [2Fe–2S]_2_-GLRX3_2_-GS_4_, once incubated with NUBP1_38–320_ and then separated, were significantly reduced in the UV–vis spectra with respect to the starting material, but their intensities were not completely bleached in the UV–vis spectra at all protein ratios and increased in intensity when moving from substoichiometric ratios to the 1:0.5 ratio (Figure 5D). Acid-labile sulfide and iron analysis on [2Fe–2S]_2_-GLRX3_2_-GS_4_, isolated from the 1:0.5 apo NUBP1_38–320_:[2Fe–2S]_2_-GLRX3_2_-GS_4_ mixture, showed that [2Fe–2S]_2_-GLRX3_2_-GS_4_ contains ∼1.2 [2Fe–2S] clusters per dimer (Table 2). Thus, ∼30% of [2Fe–2S] clusters per dimer were released from [2Fe–2S]_2_-GLRX3_2_-GS_4_ at the 1:0.5 ratio, corresponding to ∼0.5 [2Fe–2S] clusters, which is the amount experimentally determined on NUBP1_38–320_ isolated from the mixture (Table 2). This indicates that [2Fe–2S] clusters are not lost in solution during the cluster transfer reaction, but are transferred to NUBP1_38–320_. In conclusion, [2Fe–2S]_2_-GLRX3_2_-GS_4_ is able to transfer its [2Fe–2S]^2+^ clusters to the C-terminal motif by inducing NUBP1 dimerization, but no formation of a [4Fe–4S]^2+^ cluster is observed, even when 5 mM DTT was added to the mixture (Figure S4).

**Figure 6:**
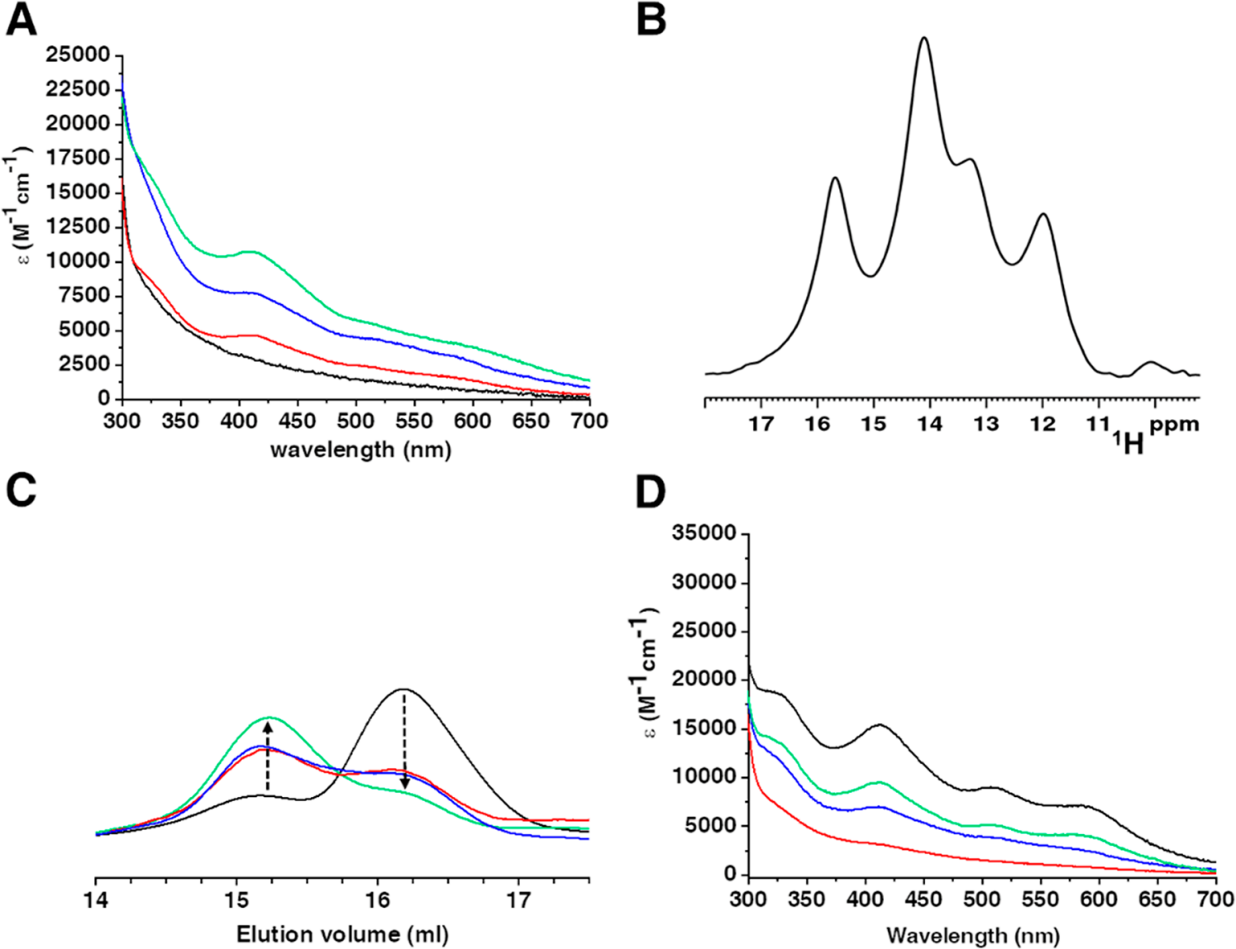
[2Fe–2S]_2_-GLRX3_2_-GS_4_ transfers [2Fe–2S]^2+^ clusters to wtNUBP1. (A) UV–vis spectra of wtNUBP1 before (black line) and after incubation with 0.5 equiv (red line), 1.0 equiv (blue line), and 1.5 equiv (green line) of [2Fe–2S]_2_-GLRX3_2_-GS_4_. (B) Paramagnetic 1D ^1^H NMR spectrum of [4Fe–4S]^2+^-wtNUBP1 obtained after reaction with 1.5 equiv of [2Fe–2S]_2_-GLRX3_2_-GS_4_ in the presence of 5 mM GSH. (C) Analytical gel filtration of wtNUBP1 before (black line) and after incubation with 0.5 equiv (red line), 1.0 equiv (blue line), and 1.5 equiv (green line) of [2Fe–2S]_2_-GLRX3_2_-GS_4_. (D) UV–vis spectra of chemically reconstituted [2Fe–2S]_2_ GLRX3_2_-GS_4_ before (black line) and after incubation with wtNUBP1 at the 0.5:1.0 (red line), 1.0:1.0 (blue line), and 1.5:1.0 (green line) [2Fe–2S]_2_-GLRX3_2_-GS_4_:wtNUBP1 stoichiometric ratios. ε values are based on dimeric protein concentration.

Different mixtures of wtNUBP1 and [2Fe–2S]_2_-GLRX3_2_-GS_4_ (0.50, 1.0, and 1.5 equiv of [2Fe–2S]_2_-GLRX3_2_-GS_4_ per monomerized wtNUBP1, with 1.5 equiv being the stoichiometric amount required to form three [4Fe–4S] clusters on dimeric wtNUBP1) were then analyzed following the same approach as that used for the two mutants, and similar conclusions were drawn. Indeed, the UV–vis and paramagnetic 1D ^1^H NMR spectra indicated a stepwise and relatively fast assembly of a [4Fe–4S]^2+^ cluster bound to the N-terminal motif of wtNUBP1 (Figure 6A and B). Analytical gel filtration showed that cluster transfer induces protein dimerization (Figure 6C), indicating that Fe/S cluster binding also occurs at the C-terminal motif of wtNUBP1, that is, the motif exclusively found to promote NUBP1 dimerization. UV–vis spectra collected on [2Fe–2S]_2_-GLRX3_2_-GS_4_, once incubated and separated from wtNUBP1 (Figure 6D), as well as acid-labile sulfide and iron analysis on wtNUBP1 and [2Fe–2S]_2_-GLRX3_2_-GS_4_ indicated an incomplete cluster transfer/assembly reaction; that is, ∼0.6 [4Fe–4S] clusters are bound to dimeric wtNUBP1 (Table 2) consistent with a [4Fe–4S] cluster assembly efficiency of ∼50% with respect to chemically reconstituted wtNUBP1. [4Fe–4S]^2+^ cluster assembly is more efficient in the presence of 5 mM DTT, a condition that gives rise to a more intense band at 410 nm (Figure S4). In the wtNUBP1:GLRX3 reaction, however, at variance with what occurs with the two mutants, the amount of released [2Fe–2S] clusters from [2Fe–2S]_2_-GLRX3_2_-GS_4_ does not correspond to the amount of Fe/S cluster bound to wtNUBP1. Indeed, ∼50% of [2Fe–2S] clusters were released from [2Fe–2S]_2_-GLRX3_2_-GS_4_ at the final 1.5:1.0 ratio(Table 2), corresponding to an expected formation of ∼1.3 [4Fe–4S] clusters. wtNUBP1 isolated from the same mixture contains, however, 0.6 [4Fe–4S] clusters (Table 2) only. The [4Fe–4S]^2+^ cluster binding lability detected at the C-terminal motif of wtNUBP1 can be considered the cause of the ∼0.7 [4Fe–4S] cluster difference. These data suggest that a [4Fe–4S]^2+^ cluster is assembled on the C-terminal motif of wtNUBP1, but, because it is not stably bound to it, it is lost upon protein isolation. The formation of a [4Fe–4S]^2+^ cluster on the C-terminal motif is confirmed by the paramagnetic 1D ^1^H NMR spectrum of wtNUBP1, isolated from the wtNUBP1 and [2Fe–2S]_2_-GLRX3_2_-GS_4_ final mixture. Indeed, a paramagnetic signal having the same chemical shift, that is, 10 ppm (Figure 6B), and intensity as that present in the paramagnetic 1D ^1^H NMR spectrum of the chemically reconstituted [4Fe–4S]^2+^ NUBP1_38–320_ (Figure 3, trace d), which exclusively contains a [4Fe–4S] cluster bound at the C-terminal motif, is detected. Moreover, the intensity of this signal is much lower than that of the signals due to the N-terminal [4Fe–4S] bound cluster (Figure 6B), according to a low cluster [4Fe–4S] occupancy at the C-terminal cluster binding site, determined by the [4Fe−4S] cluster binding lability at the C-terminal motif. This lability was detected only upon [4Fe–4S]^2+^ cluster binding. Indeed, the [2Fe–2S]^2+^ cluster bound to the C-terminal motif (i.e., in the reaction occurring between NUBP1_38–320_ and [2Fe–2S]_2_-GLRX3_2_-GS_4_) was not lost in solution, at variance with what occurs for the [4Fe–4S]^2+^ cluster (i.e., in the reaction occurring between wtNUBP1 and [2Fe–2S]_2_-GLRX3_2_-GS_4_).

To investigate the role of GSH as the reductant promoting the [4Fe–4S]^2+^ cluster assembly on wtNUBP1, we performed the reaction between [2Fe–2S]_2_-GLRX3_2_-GS_4_ and monomerized apo wtNUBP1 at the 1.5:1 stoichiometric ratio using different GSH concentrations (0, 1, 5, and 10 mM). The UV–vis spectrum, collected on wtNUBP1 isolated from the reaction mixture at 0 mM GSH, showed bands at 320, 420, 510, and 580 nm (Figure S6), which are typical of a [2Fe–2S]^2+^ cluster. By addition of up to 10 mM GSH, changes in the UV–vis spectra of wtNUBP1 isolated from the mixture were observed: the bands at 320, 510, and 580 nm decreased in intensity, and a broad unresolved band at 410 nm increased in intensity (Figure S6). These changes indicated that (i) [2Fe–2S]^2+^ clusters are transferred from GLRX3 to wtNUBP1 forming [2Fe–2S]^2+^-bound wtNUBP1 species in the absence of GSH; and (ii) the latter species is then transformed into [4Fe–4S]^2+^-bound wtNUBP1 species upon the addition of increasing amounts of the GSH reductant.

## Discussion

**Figure 7:**
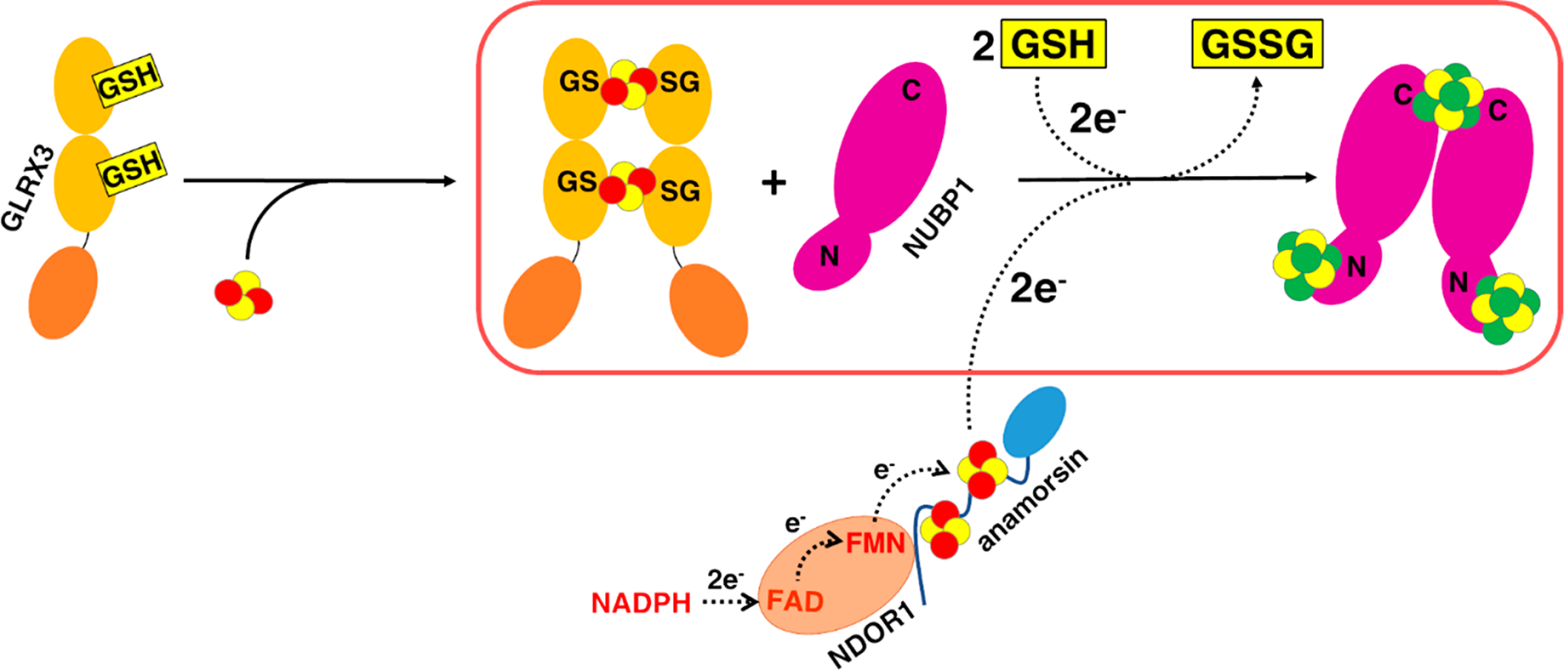
[2Fe–2S]_2_-GLRX3_2_-GS_4_ has a key role in the early stage of the CIA machinery. Our data (in the red box) showed that [2Fe–2S]_2_-GLRX3_2_-GS_4_ transfers its [2Fe–2S]^2+^ cargo to the C-terminal CPXC motif (C) and to the N-terminal CX_13_CX_2_CX_5_C motif (N) of monomeric NUBP1, promoting NUBP1 dimerization and [4Fe−4S]^2+^ cluster assembly on both N and C motifs of NUBP1. The latter process requires two electrons that can be provided by GSH and/or by the cytosolic electron transfer chain anamorsin/NDOR1. In the scheme, solid arrows indicate Fe−S cluster transfer/assembly, while dotted arrows indicate electron transfer. Fe–S clusters are represented as yellow (sulfur atoms) and red (Fe^3+^ ions) or green (delocalized Fe^2.5+^Fe^2.5+^ pairs) spheres.

In this work, we showed that [2Fe–2S]_2_-GLRX3_2_-GS_4_ is able to transfer its [2Fe–2S]^2+^ cargo to NUBP1 in its monomeric apo form (Figure 7). Although a dimeric apo state of NUBP1 was purified from *E. coli* cells, we showed that this species was induced by the presence of adventitious metal ion(s) bridging two NUBP1 molecules. Thus, it is likely that the monomeric apo NUBP1 is a potential physiologically relevant species accepting [2Fe–2S] clusters from GLRX3. Our data showed that cluster transfer from [2Fe–2S]_2_-GLRX3_2_-GS_4_ to monomeric apo NUBP1 occurs on both N-terminal and C-terminal motifs, with GLRX3 thus acting as a [2Fe–2S] cluster chaperone for both cluster binding sites. The proposed chaperone function is in agreement with the functional data available on yeast Nbp35. Indeed, it was observed that depletion of Nbp35 resulted in an increase of iron level in Grx3/4,^16^ as, when the [2Fe–2S] cluster from Grx3/4 cannot be transferred anymore to Nbp35, iron accumulates on the Grx3/4 proteins as a [2Fe–2S] cluster. A similar effect has been also observed in the ISC assembly machinery. Depletion of the ISC components Ssq1, Jac1, and Grx5, which are involved in the transfer of the [2Fe–2S] cluster from Isu1 to target proteins, leads to an accumulation of iron on Isu1, likely in the form of a [2Fe–2S] cluster.^37^ The role of GLRX3 in chaperoning [2Fe–2S] clusters to NUBP1 is also supported by other in vivo findings in yeast, showing that Nbp35 was dispensable for iron incorporation into Cfd1, and vice versa,^8,9^ suggesting that [4Fe–4S] cluster assembly on Cfd1 or Nbp35 does not depend on the other P-loop NTPase partner, but depends on Grx3/4 only.^16^

The kinetic lability observed for the C-terminal [4Fe–4S]^2+^ cluster with respect to the N-terminal [4Fe-4S]^2+^ cluster suggests a distinct functional role of the two types of clusters bound to NUBP1. As NUBP1 is involved in the formation and distribution of [4Fe–4S] clusters,^10,11^ we could speculate that the labile [4Fe–4S] cluster assembled at the C-terminal site is transferred to apo target proteins, this step being facilitated by several other assembly proteins of the CIA machinery.^5^ On the contrary, the N-terminal [4Fe–4S]^2+^ cluster might play a structural role or an electron transfer role (see later). Different roles for the two types of [4Fe–4S] clusters bound to NUBP1/Nbp35 were also supported by experimental data in yeast cells. Indeed, when yeast cells were labeled with ^55^Fe followed by exposure to a cell-permeant chelator, ∼50% of the ^55^Fe bound to Nbp35 (yeast homologous of NUBP1) was rapidly lost, whereas a nearly equal portion of ^55^Fe was more stably associated with Nbp35 for as long as 3 h in the presence of the chelator.^38^

We also showed that two [2Fe–2S]^2+^ clusters donated by GLRX3 were assembled to form a [4Fe–4S]^2+^ cluster at the N-terminal motif of NUBP1 and that its formation is independent of the presence of the C-terminal cluster motif (Figure 7). Generation of a [4Fe–4S]^2+^ cluster requires two electrons for reductively coupling the two [2Fe–2S]^2+^ clusters donated by GLRX3. In our in vitro experiments, we showed that the [2Fe–2S]^2+^ cluster coupling reaction is driven by the presence of GSH (Figure 7). We showed, indeed, that GSH was strictly required to assemble a [4Fe–4S]^2+^ cluster at the N-terminal motif, as otherwise a [2Fe–2S]^2+^ NUBP1 species was obtained in its absence. The cluster transfer/assembly process is, however, not complete in the presence of GSH and is more efficient when a reducing agent stronger than GSH, that is, DTT, although not physiological, was also present. In vivo the two required electrons could be supplied to the N-terminal site by the electron transfer chain formed by the NDOR1 and anamorsin CIA components (Figure7).^39,40^ Indeed, it has been demonstrated that the yeast homologues of NDOR1 and anamorsin, that is, Tah18 and Dre2, respectively, are specifically required for the insertion of the [4Fe–4S] cluster into the N-terminal motif of Nbp35, and human NDOR1-anamorsin can functionally replace yeast Tah18-Dre2.^9^ In support of the presence of the electron transfer chain assembling a [4Fe–4S]^2+^ cluster on the N-terminal motif of NUBP1, it was shown that a specific interaction between the anamorsin and NUBP1 homologues occurs in plants.^41,42^ In addition to the NDOR1-anamorsin electron transfer chain driving [4Fe–4S] assembly on the N-terminal motif of NUBP1, our data suggested that GSH, which is largely abundant in the cytosol, and determines a low half-cell reduction potential ranging from −200 to −240 mV,^34^ might promote the cluster coupling reaction in vivo. The observed incomplete [4Fe–4S] cluster assembly driven by GSH reductant might be determined by its higher redox potential with respect to that provided by NDOR1-anamorsin electron transfer chain. Indeed, a redox potential of −415 mV, much lower than that of GSH, was required to reduce the [2Fe–2S] cluster of Dre2,^9^ which is the terminus of the Tah18–Dre2 electron transfer chain and thus the redox center expected to provide the electrons to reductively couple two [2Fe–2S]^2+^ clusters into a [4Fe–4S]^2+^ cluster on the N-terminal site.

A GSH-driven assembly of a [4Fe–4S]^2+^ cluster via a reductive [2Fe–2S] cluster coupling process also occurs at the C-terminal motif of NUBP1 (Figure 7), although the lability of the cluster binding to the C-terminal motif determines a low [4Fe–4S] cluster occupancy at that site. In the latter reaction, cluster binding to the C-terminal motif of NUBP1 promotes protein dimerization, at variance with cluster binding to the N-terminal motif, which, indeed, does not affect the quaternary structure of NUBP1 (Figure 7). However, at variance with what is observed in the formation of the [4Fe–4S] cluster at the N-terminal motif, we found that [2Fe–2S]^2+^ clusters were not converted in a [4Fe–4S]^2+^ cluster at the C-terminal motif once the N-terminal motif was absent. This suggests a cooperativity effect between the N- and the C-terminal motifs in the formation of a [4Fe–4S] cluster at the C-terminal motif, but not in the formation of a [4Fe–4S] cluster at the N-terminal motif. It is possible that the presence of a [4Fe–4S] cluster bound at the N-terminal site is required to drive [4Fe–4S] cluster formation at the C-terminal site, but not vice versa. A possible mechanism might require that the [4Fe–4S] cluster bound at the N-terminal site would receive the electrons from the NDOR1-anamorsin electron transfer chain, and then transfer them to the C-terminal site, thus assembling a [4Fe–4S] cluster on it. In such a case, the [4Fe–4S]^2+^ N-terminal cluster is therefore playing an electron transfer role instead of a structural role. In yeast, it was proposed that the Tah18–Dre2 electron transfer chain is not required for the insertion of the [4Fe–4S] cluster into the C-terminal motif.^9^ This interpretation was based on the fact that depletion of Tah18 and Dre2 does not abolish iron incorporation on the C-terminal motif of Cfd1, but even increases iron incorporation on Cfd1. However, we have now observed that [2Fe–2S]_2_-GLRX3_2_-GS_4_ can transfer a [2Fe–2S] cluster to the C-terminal motif of NUPB1, which stably binds it without releasing it in solution. Thus, iron accumulation on Cfd1 upon depletion of Tah18 and Dre2^9^ can be now interpreted as a consequence of the lack of the electron transfer chain required for assembling a [4Fe–4S] cluster on the C-terminal motif. According to our data, Grx3/4 proteins would transfer the [2Fe–2S] cluster to Cfd1, and the [2Fe–2S] cluster remains then stacked on Cfd1 and accumulates on it in the absence of the electrons required to transform the [2Fe–2S]-bound cluster into a labile [4Fe–4S] cluster. Only the assembled [4Fe–4S] cluster, and not the [2Fe–2S] cluster, would be indeed expected to be released from the C-terminal motif in the following steps of the CIA machinery. The observed higher lability of [4Fe−4S] cluster binding with respect to [2Fe−2S] cluster binding at the C-terminal motif of NUBP1 supports this model.

## Conclusions

We propose that [2Fe–2S]_2_-GLRX3_2_-GS_4_ acts as a component of the CIA machinery at its early stage by transferring [2Fe–2S] clusters to NUBP1 and by assembling [4Fe–4S] clusters on both N-terminal and C-terminal motifs of NUBP1, which bind, respectively, a stable and a kinetic labile [4Fe–4S]^2+^ cluster. Our results allowed us to interpret and rationalize in vivo data previously reported for the components of the early stage of the CIA machinery.

## Supporting Information Available

Six figures reporting the CD and NMR spectra of wild-type NUBP1 and of NUBP1-C235A/C238A and NUBP1_38–320_ mutants; the temperature dependence of the paramagnetic 1D ^1^H NMR signals of anaerobically purified [4Fe–4S]-wtNUBP1 and [4Fe–4S]-NUBP1-C235A/C238A; the kinetic lability of the [4Fe−4S] cluster bound to chemically reconstituted NUBP1_38–320_; the comparison of the UV–vis spectra of wtNUBP1, NUBP1-C235A/C238A, and NUBP1_38–320_ before and after mixing with [2Fe–2S]_2_-GLRX3_2_-GS_4_ in the presence and in the absence of DTT; SDS-PAGE of [2Fe–2S]_2_-GLRX3_2_-GS_4_, wtNUBP1, NUBP1-C235A/C238A, and NUBP1_38–320_ after cluster transfer reaction; and the effect of GSH on cluster transfer/assembly on NUBP1 (PDF)

## References

[ref1] Lill R. Function and Biogenesis of Iron-Sulphur Proteins. Nature 2009, 460 (7257), 831–838. 10.1038/nature08301. 19675643

[ref2] Ciofi-Baffoni S.; Nasta V.; Banci L. Protein Networks in the Maturation of Human Iron–Sulfur Proteins. Metallomics 2018, 10 (1), 49–72. 10.1039/C7MT00269F. 29219157

[ref3] Maio N.; Rouault T. A. Iron-Sulfur Cluster Biogenesis in Mammalian Cells: New Insights into the Molecular Mechanisms of Cluster Delivery. Biochim. Biophys. Acta, Mol. Cell Res. 2015, 1853 (6), 1493–1512. 10.1016/j.bbamcr.2014.09.009. PMC4366362 25245479

[ref4] Braymer J. J.; Lill R. Iron-Sulfur Cluster Biogenesis and Trafficking in Mitochondria. J. Biol. Chem. 2017, 292 (31), 12754–12763. 10.1074/jbc.R117.787101. 28615445 PMC5546016

[ref5] Netz D. J. A.; Mascarenhas J.; Stehling O.; Pierik A. J.; Lill R. Maturation of Cytosolic and Nuclear Iron-Sulfur Proteins. Trends Cell Biol. 2014, 24 (5), 303–312. 10.1016/j.tcb.2013.11.005. 24314740

[ref6] Hausmann A.; Aguilar Netz D. J.; Balk J.; Pierik A. J.; Mühlenhoff U.; Lill R. The Eukaryotic P Loop NTPase Nbp35: An Essential Component of the Cytosolic and Nuclear Iron-Sulfur Protein Assembly Machinery. Proc. Natl. Acad. Sci. U. S. A. 2005, 102 (9), 3266–3271. 10.1073/pnas.0406447102. 15728363 PMC552912

[ref7] Roy A.; Solodovnikova N.; Nicholson T.; Antholine W.; Walden W. E. A Novel Eukaryotic Factor for Cytosolic Fe-S Cluster Assembly. EMBO J. 2003, 22 (18), 4826–4835. 10.1093/emboj/cdg455. 12970194 PMC212722

[ref8] Netz D. J. A.; Pierik A. J.; Stümpfig M.; Mühlenhoff U.; Lill R. The Cfd1-Nbp35 Complex Acts as a Scaffold for Iron-Sulfur Protein Assembly in the Yeast Cytosol. Nat. Chem. Biol. 2007, 3 (5), 278–286. 10.1038/nchembio872. 17401378

[ref9] Netz D. J. A.; Stümpfig M.; Doré C.; Mühlenhoff U.; Pierik A. J.; Lill R. Tah18 Transfers Electrons to Dre2 in Cytosolic Iron-Sulfur Protein Biogenesis. Nat. Chem. Biol. 2010, 6 (10), 758–765. 10.1038/nchembio.432. 20802492

[ref10] Stehling O.; Netz D. J. A.; Niggemeyer B.; Rösser R.; Eisenstein R. S.; Puccio H.; Pierik A. J.; Lill R. Human Nbp35 Is Essential for Both Cytosolic Iron-Sulfur Protein Assembly and Iron Homeostasis. Mol. Cell. Biol. 2008, 28 (17), 5517–5528. 10.1128/MCB.00545-08. 18573874 PMC2519719

[ref11] Stehling O.; Jeoung J.-H.; Freibert S. A.; Paul V. D.; Bänfer S.; Niggemeyer B.; Rösser R.; Dobbek H.; Lill R. Function and Crystal Structure of the Dimeric P-Loop ATPase CFD1 Coordinating an Exposed [4Fe-4S] Cluster for Transfer to Apoproteins. Proc. Natl. Acad. Sci. U. S. A. 2018, 115 (39), E9085–E9094. 10.1073/pnas.1807762115. 30201724 PMC6166825

[ref12] Netz D. J. A.; Pierik A. J.; Stümpfig M.; Bill E.; Sharma A. K.; Pallesen L. J.; Walden W. E.; Lill R. A Bridging [4Fe-4S] Cluster and Nucleotide Binding Are Essential for Function of the Cfd1-Nbp35 Complex as a Scaffold in Iron-Sulfur Protein Maturation. J. Biol. Chem. 2012, 287 (15), 12365–12378. 10.1074/jbc.M111.328914. 22362766 PMC3320986

[ref13] Camire E. J.; Grossman J. D.; Thole G. J.; Fleischman N. M.; Perlstein D. L. The Yeast Nbp35-Cfd1 Cytosolic Iron-Sulfur Cluster Scaffold Is an ATPase. J. Biol. Chem. 2015, 290 (39), 23793–23802. 10.1074/jbc.M115.667022. 26195633 PMC4583046

[ref14] Vitale G.; Fabre E.; Hurt E. C. NBP35 Encodes an Essential and Evolutionary Conserved Protein in Saccharomyces Cerevisiae with Homology to a Superfamily of Bacterial ATPases. Gene 1996, 178 (1–2), 97–106. 10.1016/0378-1119(96)00341-1. 8921898

[ref15] Paul V. D.; Lill R. Biogenesis of Cytosolic and Nuclear Iron-Sulfur Proteins and Their Role in Genome Stability. Biochim. Biophys. Acta, Mol. Cell Res. 2015, 1853 (6), 1528–1539. 10.1016/j.bbamcr.2014.12.018. 25583461

[ref16] Mühlenhoff U.; Molik S.; Godoy J. R.; Uzarska M. A.; Richter N.; Seubert A.; Zhang Y.; Stubbe J.; Pierrel F.; Herrero E.; Lillig C. H.; Lill R. Cytosolic Monothiol Glutaredoxins Function in Intracellular Iron Sensing and Trafficking via Their Bound Iron-Sulfur Cluster. Cell Metab. 2010, 12 (4), 373–385. 10.1016/j.cmet.2010.08.001. 20889129 PMC4714545

[ref17] Haunhorst P.; Hanschmann E.-M.; Bräutigam L.; Stehling O.; Hoffmann B.; Mühlenhoff U.; Lill R.; Berndt C.; Lillig C. H. Crucial Function of Vertebrate Glutaredoxin 3 (PICOT) in Iron Homeostasis and Hemoglobin Maturation. Mol. Biol. Cell 2013, 24 (12), 1895–1903. 10.1091/mbc.e12-09-0648. 23615448 PMC3681695

[ref18] Banci L.; Ciofi-Baffoni S.; Gajda K.; Muzzioli R.; Peruzzini R.; Winkelmann J. N-Terminal Domains Mediate [2Fe-2S] Cluster Transfer from Glutaredoxin-3 to Anamorsin. Nat. Chem. Biol. 2015, 11 (10), 772–778. 10.1038/nchembio.1892. 26302480

[ref19] Banci L.; Camponeschi F.; Ciofi-Baffoni S.; Muzzioli R. Elucidating the Molecular Function of Human BOLA2 in GRX3-Dependent Anamorsin Maturation Pathway. J. Am. Chem. Soc. 2015, 137 (51), 16133–16143. 10.1021/jacs.5b10592. 26613676

[ref20] Frey A. G.; Palenchar D. J.; Wildemann J. D.; Philpott C. C. A Glutaredoxin·BolA Complex Serves as an Iron-Sulfur Cluster Chaperone for the Cytosolic Cluster Assembly Machinery. J. Biol. Chem. 2016, 291 (43), 22344–22356. 10.1074/jbc.M116.744946. 27519415 PMC5077177

[ref21] Banci L.; Bertini I.; Ciofi-Baffoni S.; Boscaro F.; Chatzi A.; Mikolajczyk M.; Tokatlidis K.; Winkelmann J. Anamorsin Is a [2Fe-2S] Cluster-Containing Substrate of the Mia40-Dependent Mitochondrial Protein Trapping Machinery. Chem. Biol. 2011, 18 (6), 794–804. 10.1016/j.chembiol.2011.03.015. 21700214

[ref22] Patt S. L.; Sykes B. D. Water Eliminated Fourier Transform NMR Spectroscopy. J. Chem. Phys. 1972, 56 (6), 3182–3184. 10.1063/1.1677669.

[ref23] Crack J. C.; Munnoch J.; Dodd E. L.; Knowles F.; Al Bassam M. M.; Kamali S.; Holland A. A.; Cramer S. P.; Hamilton C. J.; Johnson M. K.; Thomson A. J.; Hutchings M. I.; Le Brun N. E. NsrR from Streptomyces Coelicolor Is a Nitric Oxide-Sensing [4Fe-4S] Cluster Protein with a Specialized Regulatory Function. J. Biol. Chem. 2015, 290 (20), 12689–12704. 10.1074/jbc.M115.643072. 25771538 PMC4432287

[ref24] Hagen W. R. EPR Spectroscopy of Complex Biological Iron-Sulfur Systems. JBIC, J. Biol. Inorg. Chem. 2018, 23 (4), 623–634. 10.1007/s00775-018-1543-y. 29468426 PMC6006208

[ref25] Banci L.; Camponeschi F.; Ciofi-Baffoni S.; Piccioli M. The NMR Contribution to Protein-Protein Networking in Fe-S Protein Maturation. JBIC, J. Biol. Inorg. Chem. 2018, 23 (4), 665–685. 10.1007/s00775-018-1552-x. 29569085 PMC6006191

[ref26] Bertini I.; Capozzi F.; Luchinat C.; Piccioli M.; Vila A. J. The Fe4S4 Centers in Ferredoxins Studied through Proton and Carbon Hyperfine Coupling. Sequence-Specific Assignments of Cysteines in Ferredoxins from Clostridium Acidi Urici and Clostridium Pasteurianum. J. Am. Chem. Soc. 1994, 116 (2), 651–660. 10.1021/ja00081a028.

[ref27] Li H.; Outten C. E. Monothiol CGFS Glutaredoxins and BolA-like Proteins: [2Fe-2S] Binding Partners in Iron Homeostasis. Biochemistry 2012, 51 (22), 4377–4389. 10.1021/bi300393z. 22583368 PMC3448021

[ref28] Li H.; Mapolelo D. T.; Randeniya S.; Johnson M. K.; Outten C. E. Human Glutaredoxin 3 Forms [2Fe-2S]-Bridged Complexes with Human BolA2. Biochemistry 2012, 51 (8), 1687–1696. 10.1021/bi2019089. 22309771 PMC3331715

[ref29] Haunhorst P.; Berndt C.; Eitner S.; Godoy J. R.; Lillig C. H. Characterization of the Human Monothiol Glutaredoxin 3 (PICOT) as Iron-Sulfur Protein. Biochem. Biophys. Res. Commun. 2010, 394 (2), 372–376. 10.1016/j.bbrc.2010.03.016. 20226171

[ref30] Vranish J. N.; Russell W. K.; Yu L. E.; Cox R. M.; Russell D. H.; Barondeau D. P. Fluorescent Probes for Tracking the Transfer of Iron-Sulfur Cluster and Other Metal Cofactors in Biosynthetic Reaction Pathways. J. Am. Chem. Soc. 2015, 137 (1), 390–398. 10.1021/ja510998s. 25478817 PMC4675328

[ref31] Wollers S.; Layer G.; Garcia-Serres R.; Signor L.; Clemancey M.; Latour J.-M.; Fontecave M.; Ollagnier de Choudens S. Iron-Sulfur (Fe-S) Cluster Assembly: The SufBCD Complex Is a New Type of Fe-S Scaffold with a Flavin Redox Cofactor. J. Biol. Chem. 2010, 285 (30), 23331–23341. 10.1074/jbc.M110.127449. 20460376 PMC2906325

[ref32] Gao H.; Azam T.; Randeniya S.; Couturier J.; Rouhier N.; Johnson M. K. Function and Maturation of the Fe-S Center in Dihydroxyacid Dehydratase from Arabidopsis. J. Biol. Chem. 2018, 293 (12), 4422–4433. 10.1074/jbc.RA117.001592. 29425096 PMC5868250

[ref33] Cleland W. W. DITHIOTHREITOL, A NEW PROTECTIVE REAGENT FOR SH GROUPS. Biochemistry 1964, 3, 480–482. 10.1021/bi00892a002. 14192894

[ref34] Schafer F. Q.; Buettner G. R. Redox Environment of the Cell as Viewed through the Redox State of the Glutathione Disulfide/Glutathione Couple. Free Radical Biol. Med. 2001, 30 (11), 1191–1212. 10.1016/S0891-5849(01)00480-4. 11368918

[ref35] Banci L.; Brancaccio D.; Ciofi-Baffoni S.; Del Conte R.; Gadepalli R.; Mikolajczyk M.; Neri S.; Piccioli M.; Winkelmann J. [2Fe-2S] Cluster Transfer in Iron-Sulfur Protein Biogenesis. Proc. Natl. Acad. Sci. U. S. A. 2014, 111 (17), 6203–6208. 10.1073/pnas.1400102111. 24733926 PMC4035983

[ref36] Banci L.; Bertini I.; Luchinat C. The 1H NMR Parameters of Magnetically Coupled Dimers–The Fe2S2 Proteins as an Example. Bioinorganic Chemistry: Structure and Bonding; Springer: Berlin, Heidelberg, 1990; pp 113–136.

[ref37] Mühlenhoff U.; Gerber J.; Richhardt N.; Lill R. Components Involved in Assembly and Dislocation of Iron-Sulfur Clusters on the Scaffold Protein Isu1p. EMBO J. 2003, 22 (18), 4815–4825. 10.1093/emboj/cdg446. 12970193 PMC212715

[ref38] Pallesen L. J.; Solodovnikova N.; Sharma A. K.; Walden W. E. Interaction with Cfd1 Increases the Kinetic Lability of FeS on the Nbp35 Scaffold. J. Biol. Chem. 2013, 288 (32), 23358–23367. 10.1074/jbc.M113.486878. 23798678 PMC3743505

[ref39] Banci L.; Ciofi-Baffoni S.; Mikolajczyk M.; Winkelmann J.; Bill E.; Pandelia M.-E. Human Anamorsin Binds [2Fe-2S] Clusters with Unique Electronic Properties. JBIC, J. Biol. Inorg. Chem. 2013, 18 (8), 883–893. 10.1007/s00775-013-1033-1. 23989406

[ref40] Banci L.; Bertini I.; Calderone V.; Ciofi-Baffoni S.; Giachetti A.; Jaiswal D.; Mikolajczyk M.; Piccioli M.; Winkelmann J. Molecular View of an Electron Transfer Process Essential for Iron-Sulfur Protein Biogenesis. Proc. Natl. Acad. Sci. U. S. A. 2013, 110 (18), 7136–7141. 10.1073/pnas.1302378110. 23596212 PMC3645582

[ref41] Bych K.; Netz D. J. A.; Vigani G.; Bill E.; Lill R.; Pierik A. J.; Balk J. The Essential Cytosolic Iron-Sulfur Protein Nbp35 Acts without Cfd1 Partner in the Green Lineage. J. Biol. Chem. 2008, 283 (51), 35797–35804. 10.1074/jbc.M807303200. 18957412

[ref42] Bastow E. L.; Bych K.; Crack J. C.; Le Brun N. E.; Balk J. NBP35 Interacts with DRE2 in the Maturation of Cytosolic Iron-Sulphur Proteins in Arabidopsis Thaliana. Plant J. 2017, 89 (3), 590–600. 10.1111/tpj.13409. 27801963 PMC5324674

