# Supplementary material for: GLRX3 Acts as a [2Fe–2S] Cluster Chaperone in the Cytosolic Iron–Sulfur Assembly Machinery Transferring [2Fe–2S] Clusters to NUBP1
Source: J Am Chem Soc. 2020 May 20;142(24):10794–805. doi: 10.1021/jacs.0c02266 (PMC8007109; doi:10.1021/jacs.0c02266)

Supporting Information
for

GLRX3 acts as a [2Fe-2S] cluster chaperone in the cytosolic iron-sulfur assembly machinery transferring [2Fe-2S] clusters to NUBP1

Francesca Camponeschi[1], Nihar Ranjan Prusty[1], Sabine Annemarie Elisabeth Heider[1,2], Simone Ciofi-Baffoni[1,2,]*, Lucia Banci[1,2,]*

[1]*Magnetic Resonance Center CERM, University of Florence, Via Luigi Sacconi 6, 50019, Sesto Fiorentino, Florence, Italy*

[2]*Department of Chemistry, University of Florence, Via della Lastruccia 3, 50019 Sesto Fiorentino, Florence, Italy*

E-mail: banci@cerm.unifi.it and ciofi@cerm.unifi.it

CONTENTS

Figure S1. Folding of NUBP1 is not affected by N-terminal region deletion and C235/C238 mutation.

Figure S2. Temperature dependence of paramagnetic 1D ^{1}H NMR signals of anaerobically purified wtNUBP1 and NUBP1-C235A/C238A.

Figure S3. The [4Fe-4S] cluster bound to the C-terminal motif of $NUBP1_{38\text{-}320}$ is kinetically labile.

Figure S4. Enhancement of cluster transfer and assembly process on NUBP1 in the presence of 5 mM DTT.

Figure S5. SDS-PAGE of GLRX3, wtNUBP1, NUBP1-C235A/C238A and $NUBP1_{38\text{-}320}$ after incubation between $[2Fe\text{-}2S]_2\text{-}GLRX3_2\text{-}GS_4$ and the apo NUBP1 proteins, and their separation.

Figure S6. UV-vis spectra of wtNUBP1 after incubation with $[2Fe\text{-}2S]_2\text{-}GLRX3_2\text{-}GS_4$ in the presence of increasing concentrations of GSH.

Figure S1. Folding of NUBP1 is not affected by N-terminal region deletion and C235/C238 mutation. Far UV CD (**A**) and ^{1}H 1D NMR spectra (**B**) of apo monomerized wtNUBP1, apo monomeric NUBP1 C235A/C238A and apo monomerized $NUBP1_{38\text{-}320}$.

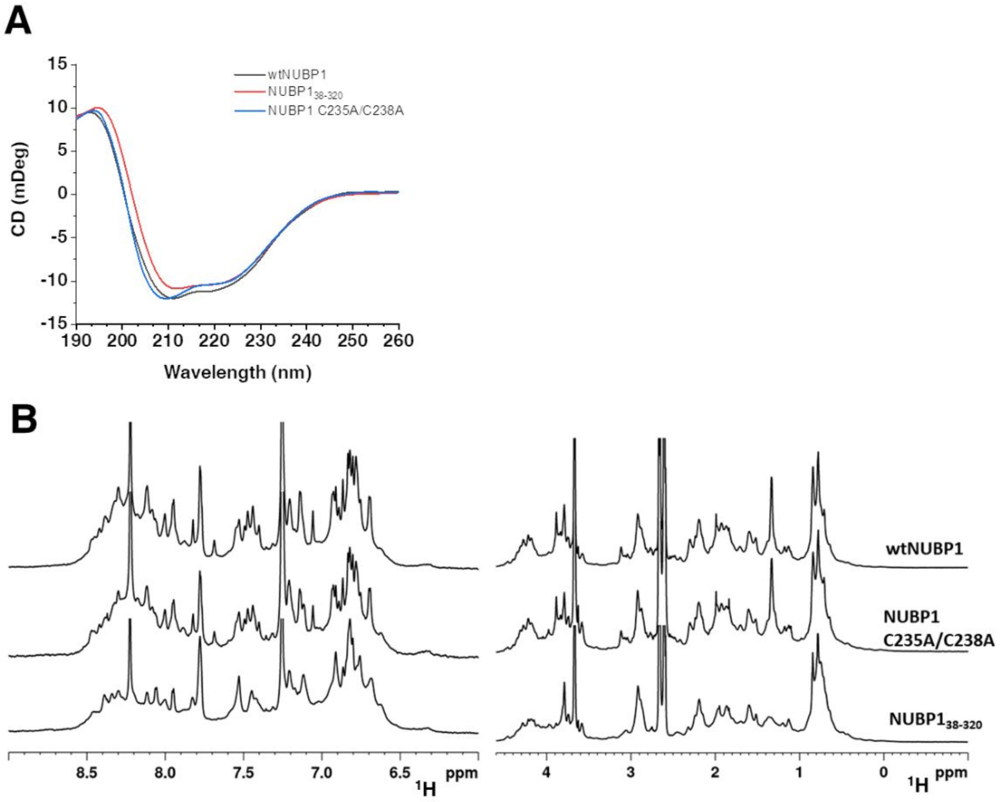

Figure S2. Temperature dependence of paramagnetic 1D ^{1}H NMR signals of anaerobically purified wtNUBP1 and NUBP1-C235A/C238A. Paramagnetic 1D ^{1}H NMR spectra of anaerobically purified (**A**) wtNUBP1 and (**B**) NUBP1-C235A/C238A recorded at 308 K, 298 K and 280 K.

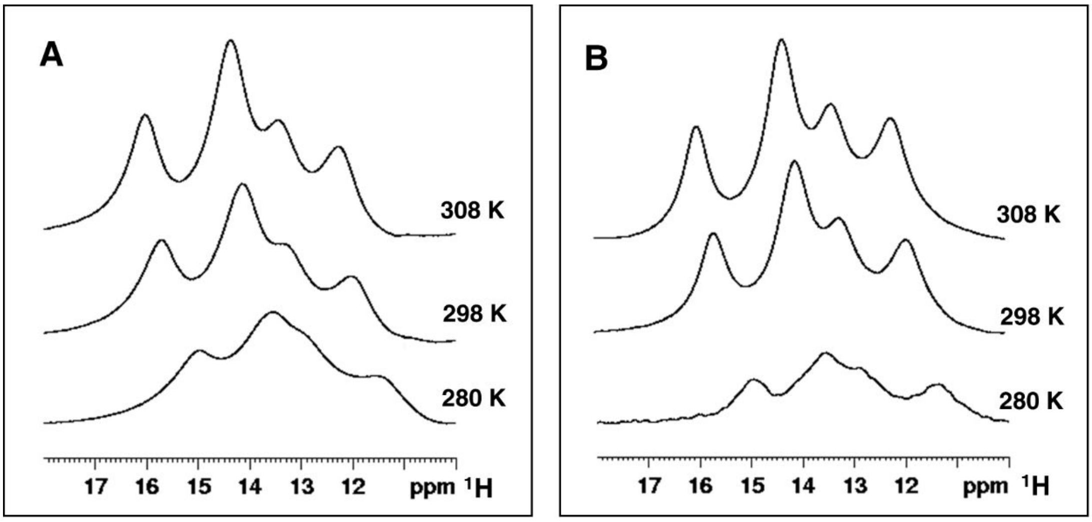

Figure S3. The [4Fe-4S] cluster bound to the C-terminal motif of NUBP1$_{38\text{-}320}$ is rapidly released/degraded. (**A**) UV-vis spectra of freshly prepared chemically reconstituted NUBP1$_{38\text{-}320}$ acquired in anaerobic conditions at different time intervals. (**B**) Kinetic curve of [4Fe-4S] cluster degradation/release obtained by plotting molar absorptivity at 410 nm of chemically reconstituted NUBP1$_{38\text{-}320}$ as a function of time. ε values are based on dimeric protein concentration.

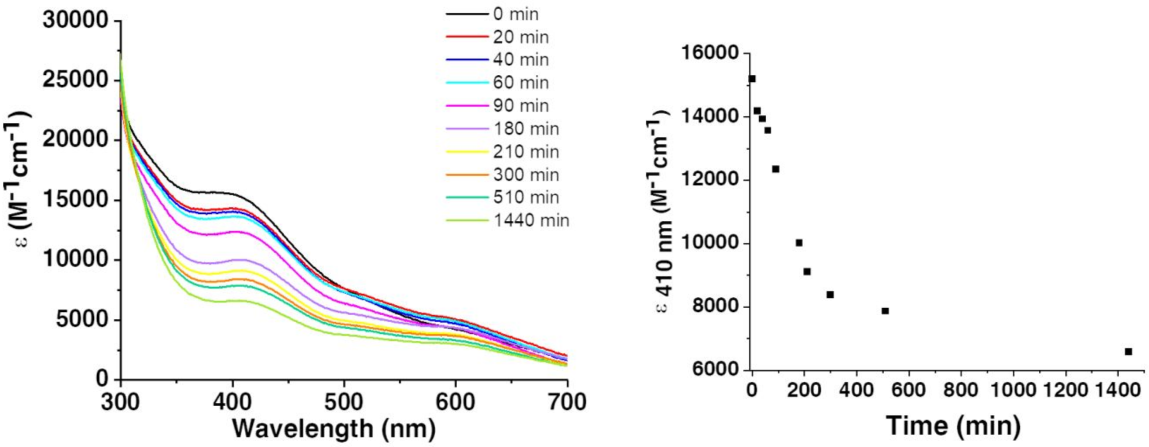

Figure S4. Enhancement of cluster transfer and assembly process on NUBP1 in the presence of 5 mM DTT. UV-vis spectra of (**A**) wtNUBP1, (**B**) NUBP1-C235A/C238A, and (**C**) $NUBP1_{38\text{-}320}$ before (black line) and after incubation with 1.5 eq, 1 eq and 0.5 eq of $[2Fe\text{-}2S]_2$ $GLRX3_2\text{-}GS_4$, respectively, in the absence (blue line) and in the presence (red line) of 5 mM DTT. (**D**) UV-vis spectra of $[2Fe\text{-}2S]_2$ $GLRX3_2\text{-}GS_4$ before (black line) and after incubation with 1 eq of NUBP1 C235A/C238A in the absence (blue line) and in the presence (red line) of 5 mM DTT. ε values are based on the monomeric protein (NUBP1-C235A/C238A) or dimeric protein (wtNUBP1, $NUBP1_{38\text{-}320}$ and GLRX3) concentration.

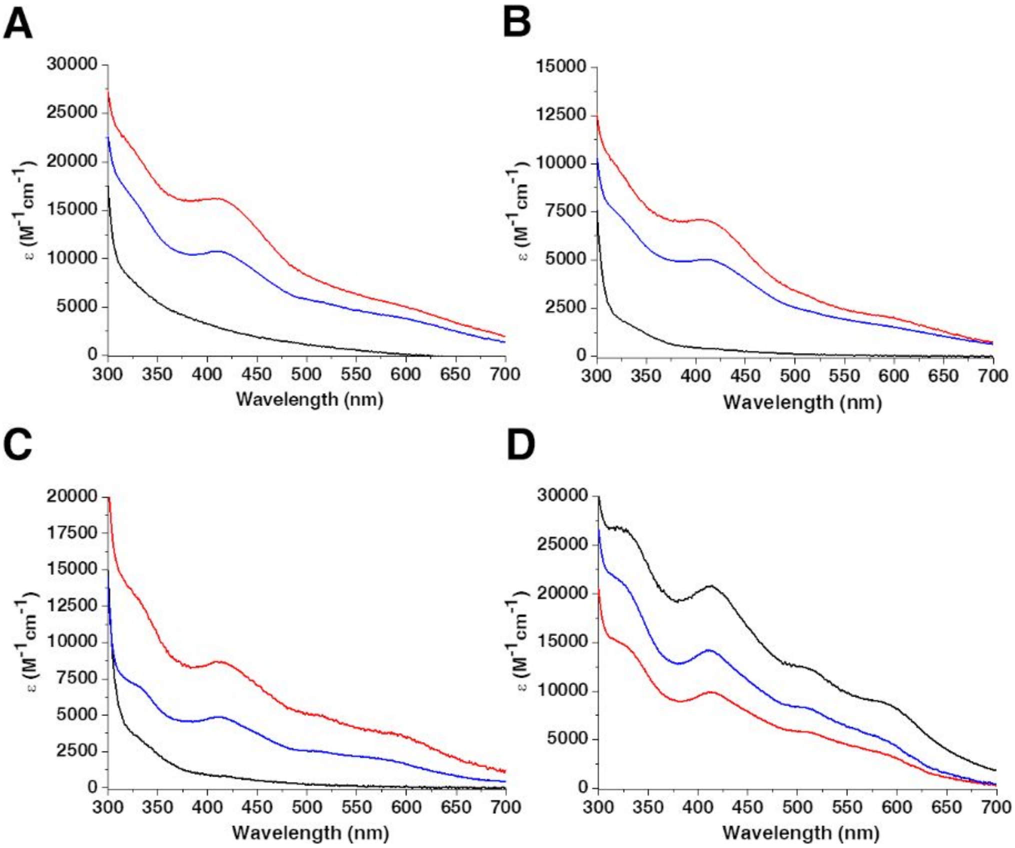

Figure S5. SDS-PAGE of GLRX3, wtNUBP1, NUBP1-C235A/C238A and $NUBP1_{38\text{-}320}$ after incubation between $[2Fe\text{-}2S]_2$-$GLRX3_2$-GS_4 and the apo NUBP1 proteins, and their separation. SDS-PAGE of $[2Fe\text{-}2S]_2$ $GLRX3_2$-GS_4 (line 1), His_6 tagged wtNUBP1 (line 2), NUBP1-C235A/C238A (line 3) and $NUBP1_{38\text{-}320}$ (line 4) once separated after incubation with 1.5 eq, 1 eq and 0.5 eq of $[2Fe\text{-}2S]_2$-$GLRX3_2$-GS_4, respectively, in the presence of 5 mM GSH.

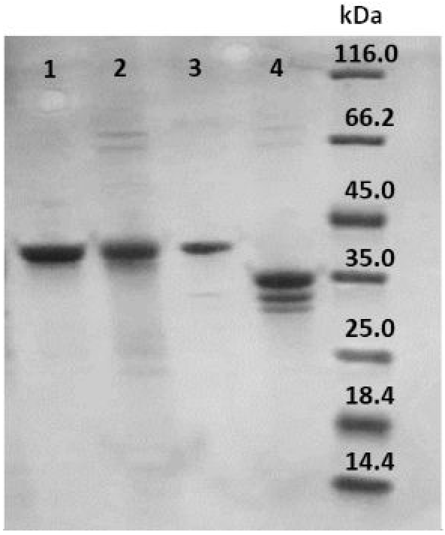

Figure S6. UV-vis spectra of wtNUBP1 after incubation with $[2Fe\text{-}2S]_2$-$GLRX3_2$-GS_4 in the presence of increasing concentrations of GSH. UV-vis spectra of wtNUBP1 before (black line) and after incubation with 1.5 eq of $[2Fe\text{-}2S]_2$-$GLRX3_2$-GS_4, in the presence of 0 mM GSH (blue line), 1 mM GSH (red line), 5 mM GSH (green line) and 10 mM GSH (magenta line).

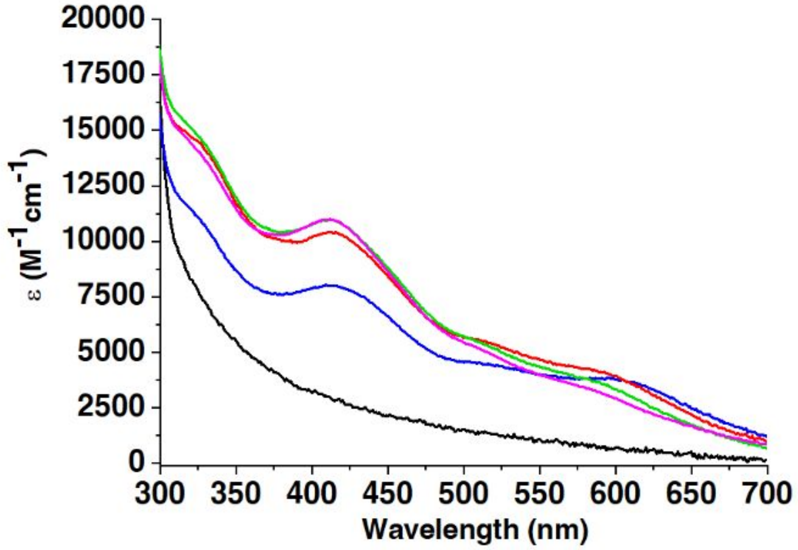

Supplement: Supplementary file 1 — ja0c02266_si_001.pdf [file ja0c02266_si_001.pdf]
